# Inheritance and enzymatic basis of anthocyanin malonylation in *Fragaria × ananassa*

**DOI:** 10.1111/tpj.70485

**Published:** 2025-10-24

**Authors:** Xiran Wang, Johanna Trinkl, Martha Wulanjati, Annika Haugeneder, Klaus Olbricht, Sonia Osorio, Iraida Amaya, José F. Sánchez‐Sevilla, Béatrice Denoyes, Aurélie Petit, Philippe Chartier, Luca Mazzoni, Bruno Mezzetti, Agnieszka Masny, Björn Usadel, Freya Ziegler, Zhen Yu, Xiaotong Zhu, Wenhao Shi, Chan Li, Chiye Yuan, Huiyun Hu, Timothy D. Hoffmann, Thomas Hoffmann, Wilfried G. Schwab

**Affiliations:** ^1^ Biotechnology of Natural Products, School of Life Sciences Technische Universität München Liesel‐Beckmann‐Str. 1 Freising 85354 Germany; ^2^ Hansabred GmbH & Co. KG Radeburger Landstr. 12 Dresden 01108 Germany; ^3^ Departamento de Biología Molecular y Bioquímica, Instituto de Hortofruticultura Subtropical y Mediterránea “La Mayora” Universidad de Málaga, Consejo Superior de Investigaciones Científicas Campus de Teatinos Málaga 29071 Spain; ^4^ Plant Breeding and Physiology, Instituto de Hortofruticultura Subtropical y Mediterránea “La Mayora” Universidad de Málaga, Consejo Superior de Investigaciones Científicas Campus de Teatinos Málaga 29010 Spain; ^5^ Centro IFAPA de Málaga, Instituto Andaluz de Investigación y Formación Agraria y Pesquera (IFAPA) Málaga 29140 Spain; ^6^ INRAE, Biologie du Fruit et Pathologie, UMR 1332, INRAE Université Bordeaux Villenave d’Ornon F‐33140 France; ^7^ INVENIO Bordeaux 33800 France; ^8^ Dipartimento di Scienze Agrarie, Alimentari e Ambientali Università Politecnica delle Marche Ancona 60131 Italy; ^9^ Department of Horticultural Crop Breeding The National Institute of Horticultural Research Konstytucji 3 Maja 1/3 Skierniewice 96‐100 Poland; ^10^ Institute of Bio‐ and Geosciences, Bioinformatics (IBG‐4) Forschungszentrum Jülich GmbH Jülich 52428 Germany

**Keywords:** *Fragaria × ananassa*, strawberry, Senga Sengana, Candonga, secondary products, anthocyanins, flavonoids, malonyltransferases, acyltransferases

## Abstract

The cultivated strawberry (*Fragaria × ananassa*) is highly valued for its attractive color and pleasant taste, which is due to the secondary metabolites it accumulates. To clarify which factors influence the content of secondary metabolites in the fruit, F1 hybrids of the commercial strawberry cultivars “Senga Sengana” and “Candonga,” which are adapted to different climatic zones, were produced. F1 progeny were grown in five European countries, and the content of secondary metabolites was analyzed. The results show that the genetic factor plays a more important role compared with environmental conditions. Cultivar “Senga Sengana” produced significantly more pelargonidin‐3‐O‐(6′‐O‐malonyl)glucoside than “Candonga,” regardless of the environmental conditions, and the progeny segregated with a ratio of 1:1 in this trait. Twenty‐four putative malonyltransferase genes were cloned from *F. × ananassa* (*FaMAT*) “Senga Sengana” and “Candonga,” and six FaMATs showed enzymatic activity. Three FaMAT enzymes are promiscuous and malonylate flavonoid glucosides and anthocyanins, whereas the others are specific for quercetin‐3‐*O*‐glucoside and kaempferol‐3‐*O*‐glucoside. Expression data indicate that the gene products of *FaMAT1* and *4* catalyze the malonylation of anthocyanins in “Senga Sengana” and the related hybrids, while the corresponding functional alleles in “Candonga” and the second group of hybrids are only slightly expressed in the mature fruit. Thus, altered *FaMAT* transcription genetically determines the different levels of malonylated anthocyanins in the progeny.

## Significance Statement

Fruits from various cultivars of the cultivated strawberry (*Fragaria × ananassa*) exhibit significant variation in the levels of malonylated anthocyanins, which is attributed to the differential expression of malonyltransferase genes, whose products demonstrate acceptor substrate promiscuity. Malonylation facilitates the accumulation of higher quantities of phenols in the fruit.

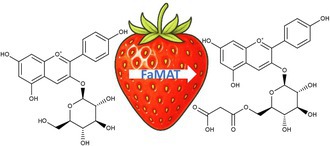

## INTRODUCTION

Strawberries (*Fragaria × ananassa*, 2*n* = 8*x* = 56) are known worldwide for their attractive color, pleasant flavor, and health benefits. Anthocyanins give strawberries their appealing red color but also have antioxidant properties (Castillejo et al., 2020; Da Silva et al., 2007; Dzhanfezova et al., 2020; Tena et al., 2020; Yoshida et al., 2002). At the same time, these secondary products protect plants against ultraviolet radiation and other biotic and abiotic stress factors (Cui et al., 2023; Kovinich et al., 2015; Wen et al., 2020). In 2024, over 8 million tons of octoploid cultivated strawberries were grown worldwide (Feng et al., 2021), with the composition of the fruit differing considerably between the various strawberry cultivars (Nowicka et al., 2019). Furthermore, the production and nutritional values of strawberries vary greatly depending on the growing conditions, including temperature, humidity, and photoperiod (Cervantes et al., 2020; Li et al., 2020). Several studies have investigated the extent to which these factors influence the accumulation of secondary products (Akhatou et al., 2016; Carbone et al., 2009; Haugeneder et al., 2018; Josuttis et al., 2013; Palmieri et al., 2017; Tulipani et al., 2011). The results of the studies illustrate the major impact of developmental processes on metabolite concentrations and show genotype‐dependent differences as well as the influence of the environment on (pro)anthocyanidin and flavonoid metabolism.

Flavonoids and anthocyanins are downstream metabolites of the phenylpropanoid pathway, with the product of flavanone 3‐hydroxylase (F3H), dihydrokaempferol, being the branch point (Sendri & Bhandari, 2024). Other essential enzymes are flavonoid‐3′‐hydroxylase (F3′H), dihydroflavonol 4‐reductase (DFR), anthocyanidin synthase (ANS), flavonoid‐3′,5′‐hydroxylase (F3′5′H), and flavonol synthase (FLS). UDP‐sugar‐dependent flavonoid glucosyltransferase (UFGT) catalyzes glycosylation, which improves the stability and solubility of the compounds. Transcription factors regulate the expression of biosynthetic genes. Important here is a ternary MYB‐bHLH‐WD40 (MBW) complex, which consists of FaMYB5 from the R2R3 family, FaEGL3, and FaLWD1, and controls flavonoid biosynthesis (Jiang et al., 2023). Several other members of the R2R3‐MYB family are also involved in regulating branches of flavonoid biosynthesis in *F. × ananassa*, such as FaMYB1, 9, 10, and 11 (Wang et al., 2020). The stable anthocyanins and flavonoids are stored in the vacuole of strawberry fruit cells, mainly pelargonidin‐3‐glucoside (Pg‐3‐glc), cyanindin‐3‐glucoside (Cy‐3‐glc), kaempferol‐3‐glucoside (K‐3‐glc), and quercetin‐3‐glucoside (Q‐3‐glc). Detailed metabolome studies showed that these compounds are available for the synthesis of more complex secondary products. Examples are pelargonidin‐3‐*O*‐(6′‐*O*‐malonyl)glucoside (Pg‐3‐MG), pelargonidin‐3‐*O*‐(6′‐*O*‐acetyl)glucoside, pelargonidin‐3‐*O*‐(6′‐*O*‐succinyl)glucoside, and pelargonidin‐3‐rutinoside (Bakker et al., 1994; Da Silva et al., 2007; Hong & Wrolstad, 1990). Pg‐3‐MG was identified as one of the major pigments in some Japanese strawberry cultivars, accounting for 5–24% of total anthocyanins, but two cultivars contained no malonylated anthocyanins at all (Yoshida et al., 2002). Numerous quantitative trait loci (QTL) mapping studies have been conducted to study various aspects of fruit quality of cultivated strawberries (*F. × ananassa*) and the woodland strawberry (*F. vesca*), including the identification of QTL for total anthocyanin content (Labadie et al., 2020; Labadie et al., 2022; Lerceteau‐Köhler et al., 2012; Urrutia et al., 2016; Vallarino et al., 2019; Verma et al., 2017). The presence and absence of Pg‐3‐MG was found to be under the control of a major locus on linkage group LG6b on the *F. × ananassa* linkage maps (Davik et al., 2020). However, the candidate gene, enzyme, or transcription factor underlying this QTL has not yet been characterized.

Malonyltransferases (MATs), which are involved in polyphenol synthesis, are members of the BAHD acyltransferase superfamily that have HXXXD, YFGNC, and DFGWG motifs in their protein sequences and catalyze the transfer of malonyl groups from the donor malonyl‐CoA to various substrates (Moghe et al., 2023). Plant MATs can be divided into two groups according to their function. Group 1 comprises MATs that contribute to oil storage and are components of the fatty acid synthase system (Malonyl‐CoA‐Acyl carrier protein Transacylase) such as *Allium porrum* L. MCAT (Lessire & Stumpe, 1983), Arabidopsis MCAT (Guan et al., 2020), and Brassica napus MCAT (Qu et al., 2014). Group 2, to which the BAHD acyltransferases belong, catalyze the acylation of secondary products such as anthocyanins and flavonoid glycosides, but also of phytohormone glycosides, thus influencing plant development. Arabidopsis phenolic glucoside MAT1 (PMAT1) regulates metabolic homeostasis, causes dwarfism, delays flowering, and reduces fertility (Gan et al., 2021). Ss5MaT1 from scarlet sage (*Salvia splendens*) was the first plant MAT shown to malonylate anthocyanins (Suzuki et al., 2001), producing Pg‐3‐MG and showing a strong acyl donor preference for malonyl‐CoA. In the model plant Arabidopsis, At5MAT catalyzes the corresponding reaction (D'Auria et al., 2007). To date, 35 members of the BAHD‐MAT have been identified, most of which exhibit high selectivity with respect to the acceptor substrate. In contrast, the recently discovered MAT1 from *Cistanche tubulosa* is more tolerant to the acceptor (Liu et al., 2024). The cultivated strawberries and wild strawberries also accumulate malonylated anthocyanins, which are presumably formed by the gene products of MATs that have already been detected in the genomes of various *Fragaria* genotypes (Davik et al., 2020). Biochemical evidence has not yet been provided.

In this study, we aimed to elucidate the results of a comparative metabolite profiling analysis of strawberry fruit from 64 individuals of a crossbred population (GoodBerry project) at the molecular level. The analyses revealed that approximately half of the F1 progeny exhibited high levels of malonylated anthocyanins, similar to the parent *F. × ananassa* “Senga Sengana,” while the other half showed low levels, akin to the second parent “Candonga,” regardless of the climatic conditions of their growing region. Driven by transcriptome data, we cloned, sequenced, and characterized 24 *FaMAT* candidate genes. Two were identified as pseudogenes, eight were inactive with the provided substrates, and nine utilized quercetin and kaempferol glucoside as acceptors. Among these, FaMAT1C (from “Candonga”), FaMAT1S (from “Senga Sengana”), and FaMAT4C_1_/4S_1_ (from both cultivars) were capable of malonylating anthocyanins. Since all functional FaMATs were more highly expressed in fully ripe fruit in “Senga Sengana” compared with in “Candonga,” we conclude that FaMAT1C, FaMAT1S, and FaMAT4C_1_/4S_1_ are responsible for the production of Pg‐3‐MG and Cy‐3‐MG during fruit ripening.

## RESULTS

### The genotype influences the metabolite content in the fruits of the Goodberry population more than the environment

**Figure 1:**
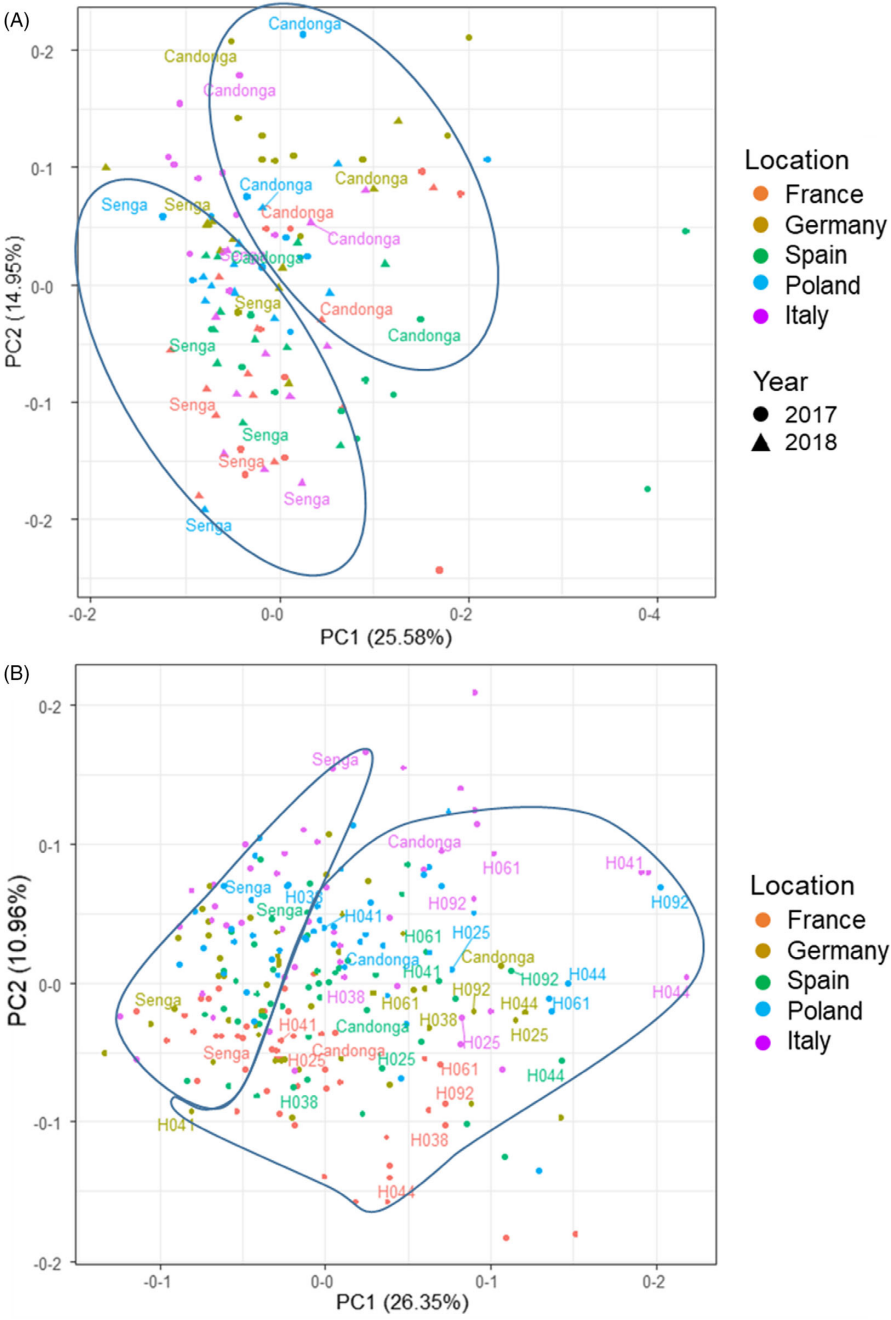
Principal component analysis (PCA) of the metabolite content in selected genotypes of the GoodBerry population. (A) Harvest years 2017 and 2018. Eleven genotypes were harvested at all locations in both years. The symbols represent the different genotypes. The parents Senga = “Senga Sengana” and “Candonga” are highlighted. The coloring distinguishes the locations; the shape of the symbols indicates the harvest years. (B) Harvest year 2018 (*n* = 48 genotypes). The genotypes H025, H038, H041, H044, H061, and H092 are selfing lines of “Candonga.” The dots represent the different genotypes, and the colors represent the different locations.

In the Goodberry project, the pseudo full‐sibling F1 population of *F. × ananassa* obtained from the cross between “Candonga” and “Senga Sengana” was grown together with the parent lines at five different locations in Europe (Germany, Poland, France, Spain, and Italy) in 2017 and 2018 (Table S1). The non‐volatile metabolites in the fruit of the progeny were analyzed by LC–MS, and their content was expressed as relative concentration (parts per million, ppm) compared with an internal standard (IS), assuming a response factor of one (ppm equ. IS) (Table S2; Data S1). The subset of the GoodBerry cross‐population studied in this work comprised 64 genotypes, including the parents “Senga Sengana” and “Candonga” (Figure S1). However, due to plant propagation problems and crop failures, only fruit from 11 genotypes from both years and all locations could be analyzed across the board (Figure S1). In the first year, 12 genotypes at all locations bore sufficient fruit for analysis, while in the second harvest year, the number of genotypes from all locations amounted to 48, including six selfing lines of “Candonga” (Figure S1). The metabolite concentration of the 11 genotypes of the crossing population that were quantified in both 2017 and 2018 (Figure 1A) and the 48 genotypes that were analyzed by LC–MS in the 2018 harvest year (Figure 1B) were subjected to a principal component analysis (PCA) to determine the influence of the genotype and the environment on the metabolite contents. It was primarily the genotype that led to the separation of the samples, as the parent cultivars, for example, always arranged themselves separately from each other (Figure 1A,B). Moreover, the “Candonga” selfing lines (Figure 1B) scatter around the respective “Candonga” values. On average, they tend to have more extreme values than “Candonga,” but their relationship to “Candonga” is clearly recognizable in comparison to “Senga Sengana.” In addition to the influence of the genotype, an effect of the environment could also be determined, which can be seen in the scattering of the metabolite contents for the respective genotypes at the different locations, that is, samples with the same color code are closer together (Figure 1).

### Flavanols and malonylated anthocyanins show a strong genotype effect

**Figure 2:**
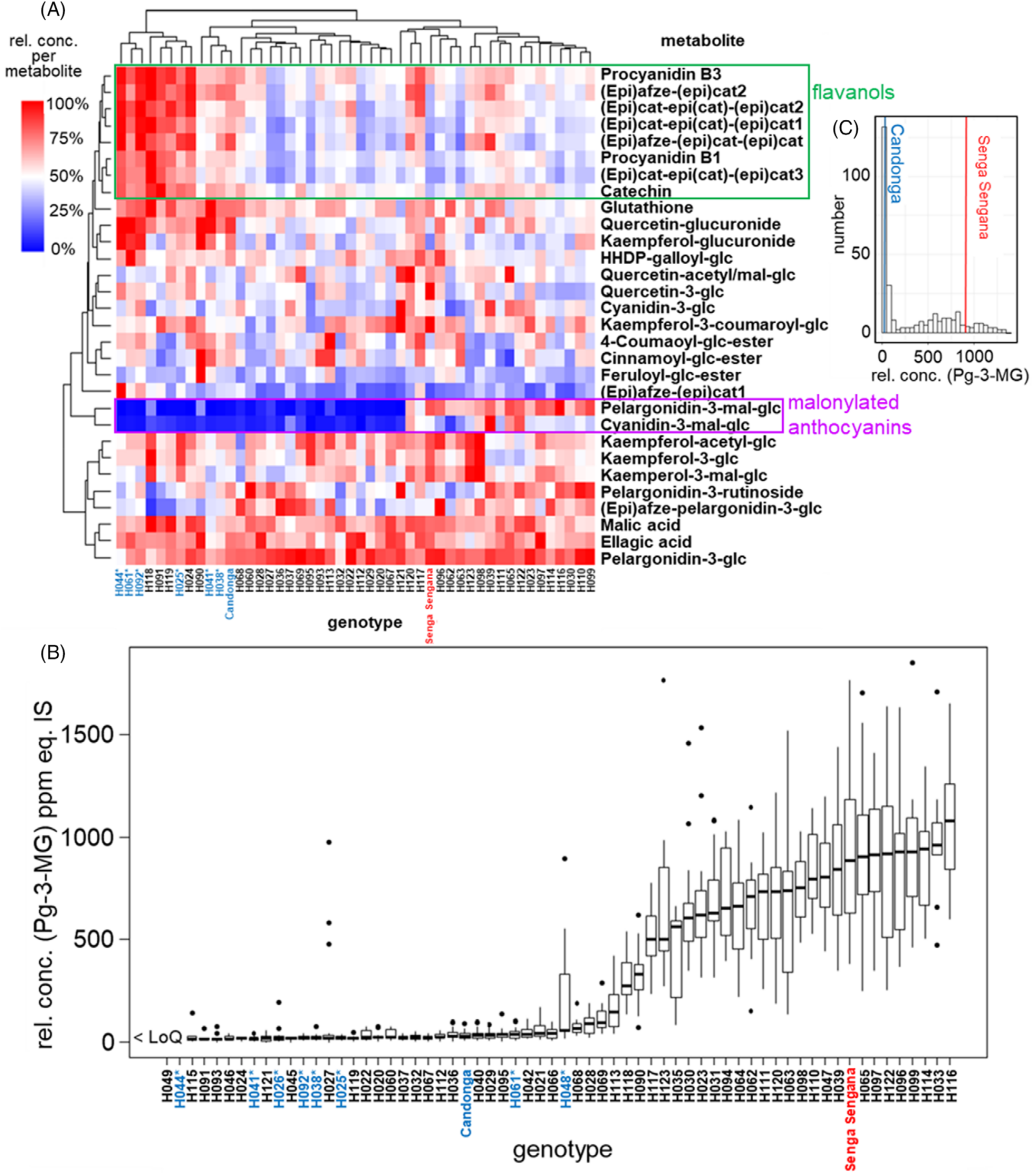
Segregation of the progeny of the GoodBerry population based on the concentration of malonylated anthocyanins. (A) Heatmap of the mean relative metabolite contents across all locations in fruit of 48 genotypes harvested in 2018. 100% corresponds to the highest relative concentration of an analyte in the data set. * = “Candonga” selfing lines and “Candonga” are shown in blue. “Senga Sengana” is shown in red. (B) Boxplots of the pelargonidin‐3‐malonylglucoside (Pg‐3‐MG) contents of the fruit of the GoodBerry genotypes in 2018. The values for the 64 individual genotypes were averaged across all available locations. “Candonga” and its six selfings are marked in blue whereas the other parent “Senga Sengana” is marked in red. For two genotypes, the concentrations were below the limit of quantification (LoQ). (C) Histogram of the Pg‐3‐MG concentrations of the GoodBerry genotypes in the 2018 harvest year. The mean concentrations of the two parent cultivars are marked.

To take a closer look at the influence of the genotype, the metabolite concentrations in the fruit of 48 genotypes harvested in 2018 were averaged across all locations and presented as a heatmap (Figure 2A). High concentrations of the respective metabolites in the fruit of individual genotypes are displayed in red (>50% of the highest relative concentration) and low levels in blue (<50% of the relative concentration). Particularly high flavanol contents were found in the fruit of the genotypes H024, H044, H091, and H118 (Figure 2A). Interestingly, the fruit of “Candonga” always showed high flavanol contents, while the fruit of “Senga Sengana” had lower flavanol concentrations, similar to H027, H036, and H063. However, one of the most apparent genotype effects concerned the anthocyanins, specifically the malonylated anthocyanins Pg‐3‐MG and Cy‐3‐MG. The highest mean Pg‐3‐MG content was 1085 ± 156 ppm equ. IS, and the respective Cy‐3‐MG content was 117 ± 17 ppm equ. IS. Based on the limit of quantification (LoQ), this means a factor of over 50 for Cy‐3‐MG and over 100 for Pg‐3‐MG between the genotype with the highest and lowest content (Figure 2B).

The histogram showing the frequencies of genotypes with a defined Pg‐3‐MG concentration revealed a bimodal distribution suggesting a Mendelian genetic control (Figure 2C). In the low concentration range, “Candonga” appeared with 38 ± 17 ppm equ. IS, and “Senga Sengana” with 903 ± 246 ppm equ. IS was found in the high concentration range (Figure 2B,C). In fruit of 37 genotypes of the GoodBerry population, including “Candonga” and its selfings, low levels of malonylated anthocyanins were quantified (relative concentration of Pg‐3‐MG less than 200 ppm equ. IS), while fruit of 26 genotypes, including “Senga Sengana,” showed higher levels of malonylated anthocyanins. The individuals of the F1 progeny fitted a 1:1 ratio, and all “Candonga” selfings were similar to their parent. These results suggest genetic control of the relative concentration of Pg‐3‐MG by a single locus, with “Candonga” being homozygous and “Senga Sengana” heterozygous for this locus. A genotype dependency was also found for the other anthocyanins, but it was not nearly as pronounced as for the malonylated anthocyanins. In the case of pelargonidin‐3‐rutinoside (Pg‐3‐rut), the highest mean concentration was 602 ± 168 ppm equ. IS (H060), and the lowest 112 ± 43 ppm equ. IS (H118). By comparing the total and individual amounts of pelargonidin metabolites in the progeny fruit, we concluded that the formation of Pg‐3‐MG facilitates the accumulation of larger amounts of pelargonidin metabolites (Figure S2).

### Pelargonidin‐3‐glucoside is preferentially malonylated by the “Senga Sengana” group

Based on the content of malonylated anthocyanins in the fruit of the progeny, these can be divided into a “Candonga” and a “Senga Sengana” group with low and high concentrations of the malonylated derivatives, respectively (Figure 2B).

**Figure 3:**
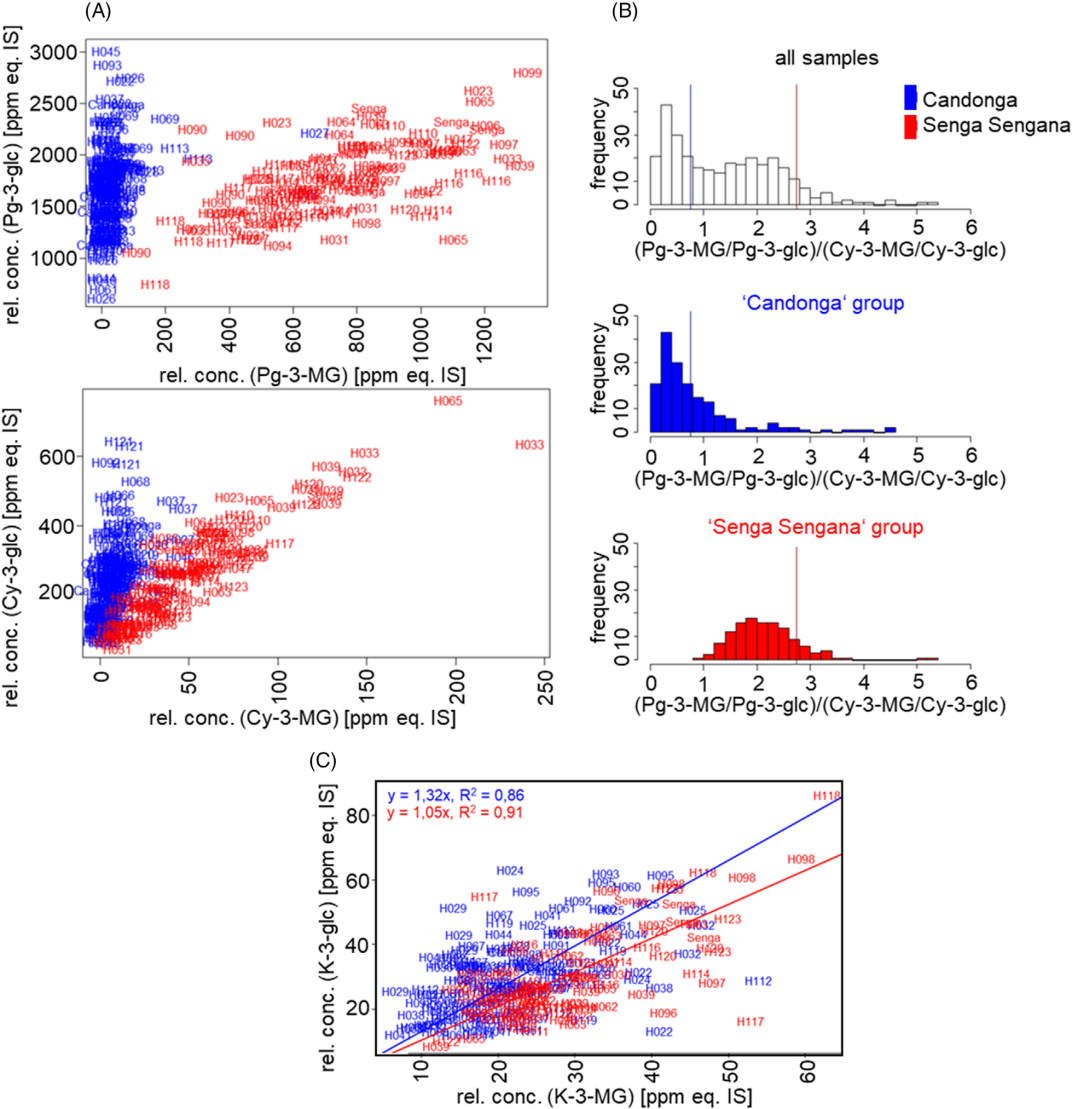
Dependence of the concentrations of pelargonidin and cyanidin derivatives of the GoodBerry population. (A) Scatter plots showing the relationship between the concentration of malonylated anthocyanins (Pg‐3‐MG and Cy‐3‐MG) and anthocyanins (Pg‐3‐glc and Cy‐3‐glc). The values for the 64 individual genotypes in 2018 were averaged across all available locations. (B) Histograms of the frequency of progeny differing in conversion rates between Pg‐3‐glc and Cy‐3‐glc; blue: “Candonga” group, red: “Senga Sengana” group. (C) Scatter plot showing the relationship between the concentration of malonylated kaempferol‐3‐glucoside (K‐3‐MG) and kaempferol‐3‐glucoside (K‐3‐glc). Slope and R^2^ of the regression line for the blue (“Candonga” group) and red (“Senga Sengana” group) were calculated.

Since the concentrations of Pg‐3‐Mg and Cy‐3‐MG in the progeny behaved similarly, a correlation analysis indicated a strong dependence between the two, independent of environmental conditions. A dependence of the levels of the glucosides Pg‐3‐glc and Cy‐3‐glc and their malonylated derivatives Pg‐3‐MG and Cy‐3‐MG was also found, which divides the progeny into two groups (Figure 3). At identical concentrations of Pg‐3‐glc (or Cy‐3‐glc), the blue‐colored group of individuals and selfings (“Candonga” group) produces less Pg‐3‐MG (or Cy‐3‐MG) than the red‐colored group (“Senga Sengana” group) (Figure 3A). Accordingly, the enzymatic *in planta* malonylation activity of the “Candonga” group should be lower than that of the “Senga Sengana” group. By calculating the conversion rate (ratio of malonylated to non‐malonylated anthocyanin) and comparing the corresponding values of the pelargonidin pair with those of the cyanidin pair, the ratio of *in vivo* activities can be derived (Figure 3B). It was found that the “Senga Sengana” group converts Pg‐3‐glc better than the “Candonga” group compared with Cy‐3‐glc, which can be inferred from the higher values for the ratio (Pg‐MG/Pg‐3‐glc)/(Cy‐3‐MG/Cy‐3‐glc) (Figure 3B). A dependence of the contents of glucosides and malonylated glucosides could also be determined for the flavonols kaempferol and quercetin, but in contrast to the anthocyanins (Figure 3A), no clear separation into genotype groups was recognizable (Figure 3C). A closer look reveals a small difference. Comparing the respective linear regression lines of the correlation plots with origin at zero, a slightly steeper increase, that is, a slightly lower conversion for the “Candonga” group is noticeable (Figure 3C). This is consistent with the lower malonyltransferase activity toward anthocyanins of this genotype group compared with the “Senga Sengana” group.

### Homologous malonyltransferase genes are expressed in fruit of *F. Vesca*

Different malonyltransferase activity was assumed to be the cause of the segregation of the crossing population into a group with high and a group with low levels of malonylated anthocyanins. The gene annotation of the *Fragaria vesca* genome (v1.0 hybrid), the only *Fragaria* genome sequence available at the beginning of the study, contains potential candidate genes for malonyltransferases (MAT) (Tennessen et al., 2014) (Table S3). To assess their importance for the malonylation of anthocyanins during strawberry fruit ripening, the respective transcript levels of the candidate genes in different *F. vesca* cultivars were first analyzed using an in‐house generated transcriptome dataset (Härtl et al., 2017). For 10 of the 14 selected candidate *MAT* genes annotated as potential anthocyanin acyltransferases, transcription was detected in the ripe fruit of *F. vesca* cultivars (Figure S3). The target gene(s) should be expressed in the receptacle. Four of the 10 transcribed genes were mainly expressed in the achenes. Anthocyanins are only formed at the end of fruit ripening, which is why gene expression for the anthocyanin *MAT* candidate gene is expected at the ripe fruit stage. Gene 29 348 was expressed neither in the achenes nor in the receptacle of the ripe fruit (Figure S3). To determine the cause of the segregation of potential *MAT* genes within the GoodBerry population, the *MAT* candidate genes 04261 (MAT1), 04262 (MAT2), 03835 (MAT3), 29 347 (MAT4), and 04266 (MAT5) of the two parents “Senga Sengana” and “Candonga” were isolated, transferred into *E. coli*, and the recombinant proteins were produced (Table S3).

### Orthologous MAT genes of *F. Vesca* were isolated from *F. × ananassa* cultivars

All candidate genes amplified from the isolated DNA of the parents “Senga Sengana” and “Candonga” were free of introns and could be successfully isolated from the parents using primers generated from the *F. vesca* sequences (Table S4; Figure S4). The gene and protein sequences isolated from “Senga Sengana” and “Candonga” differed (Figure S5). The potential MAT obtained is referred to below as MAT1 to MAT5, whereby the alleles from “Candonga” are abbreviated as “C” (1C to 5C) and from “Senga Sengana” as “S” (1S–5S). Two of the 10 genes showed a premature stop codon in the “Senga Sengana” sequences (2S, 4S) (Figure S5).

### Three MAT show enzymatic activity toward anthocyanins and flavonol glucosides

**Figure 4:**
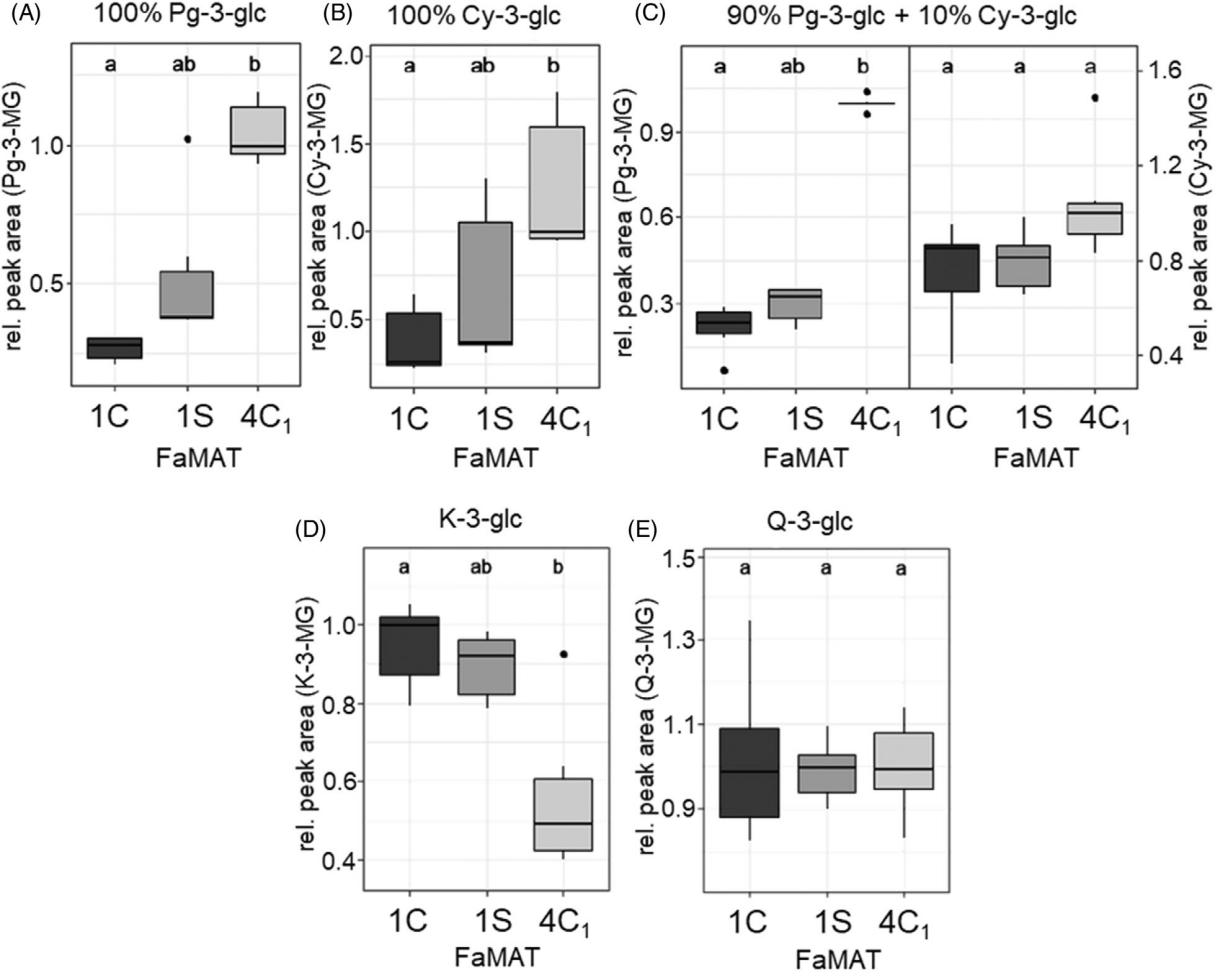
Relative product formation of MAT from “Candonga” (FaMAT1C and 4C_1_) and from “Senga Sengana” (FaMAT1S). Anthocyanins and flavonol glucosides served as acceptor substrates. (A) substrate Pg‐3‐glc (100%); (B) substrate Cy‐3‐glc (100%); (C) substrate Pg‐3‐glc (90%) and Cy‐3‐glc (10%); (D) substrate K‐3‐glc; (E) substrate Q‐3‐glc. Letters (a and b) indicate significant differences (Dunn–Bonferroni test, *P* ≤ 0.05). The relative peak areas of the products are shown in relation to the largest peak area median of a product that was set to 1.

Eight of the 10 genes expected to encode active MAT were introduced into *E. coli*; the recombinant proteins were produced and affinity purified using the glutathione transferase (GST) tag of the fusion protein (Figure S6). The purified recombinant proteins were incubated with acyl‐acceptor substrates and malonyl‐CoA as the acyl donor at pH 7 and 30°C for 16 h. The identity and purity of the proteins were verified by SDS‐PAGE (Figure S6). The anthocyanins Pg‐3‐glc and Cy‐3‐glc, and the flavonols K‐3‐glc and Q‐3‐glc were tested as acyl‐acceptor substrates. All of these compounds are natural metabolites of strawberries, the malonylated forms of which have already been detected in strawberry plants (Hanhineva et al., 2008). The pGEX‐4 T‐1 empty vector protein expression in *E. coli* BL21 served as a negative control. Since no reference material was available for any of the products, the relative peak area was considered for the quantitative comparison of the enzyme activities. The identity of the products was verified by means of fragmentation patterns and comparison with a strawberry fruit extract. The quantification of the products was carried out using the UV spectrum (anthocyanins at 520 nm and flavonol glucosides at 280 nm). Three of the tested FaMAT candidates, namely, FaMAT1C and FaMAT4C_1_ from “Candonga,” as well as FaMAT1S from “Senga Sengana,” showed catalytic activity with the tested substrates (Figure 4).

FaMAT4C_1_ showed significantly higher activities for the conversion of both Pg‐3‐glc and Cy‐3‐glc compared with FaMAT1C and FaMAT1S, with FaMAT1S being more catalytically active than FaMAT1C. In addition to the activity against a single substrate, the simultaneous conversion of the two substrates was also tested for the anthocyanins. The ratio of Pg‐3‐glc and Cy‐3‐glc was simulated to the ratio in the strawberry fruit of approx. 9:1 (Da Silva et al., 2007). Again, a significantly higher activity of FaMAT4C_1_ compared with FaMAT1C was found. FaMAT1S preferentially converted Pg‐3‐glc compared with FaMAT1C, but no significant difference was found for Cy‐3‐glc. FaMAT4C_1_ formed more Cy‐3‐MG. Similar to the metabolite profile of the GoodBerry population, the ratio between the two analyte groups was calculated based on the product to reactant ratio. The ratio of Pg‐3‐MG/Pg‐3‐glc to Cy‐3‐MG/Cy‐3‐glc was 0.11 ± 0.05 for FaMAT1C, 0.14 ± 0.02 for FaMAT1S, and 0.31 ± 0.13 for FaMAT4C_1_, indicating that Pg‐3‐glc was best metabolized with FaMAT4C_1_ compared with Cy‐3‐glc. FaMAT1C malonylated more Cy‐3‐glc compared with Pg‐3‐glc during simultaneous conversion.

In addition to anthocyanins, flavonol glucosides were also used as acceptor substrates of the MAT (Figure 4). In contrast to the high conversion rate for anthocyanins, FaMAT4C_1_ showed a low enzymatic activity for K‐3‐glc compared with the other two MATs. FaMAT1C and FaMAT1S showed similar activities for K‐3‐glc. Q‐3‐glc was the only substrate tested that was converted equally well in the enzyme assay with all three FaMATs. Since the reactant Q‐3‐glc showed about half the intensity after the reaction compared with the blank control, that is, half of the acceptor substrate was converted and the acceptor and donor substrates were initially added in a 2:1 ratio, it can be assumed in this case that all of the malonyl‐CoA was consumed.

### Transcriptome analysis of GoodBerry genotypes provides further MAT candidates

Since the results obtained could not clearly explain the metabolite data, we continued to characterize further MAT genes from *Fragaria*. Thirty‐two selected genotypes of the GoodBerry population were subjected to transcriptome analysis and the transcript abundance (transcripts per million reads in red versus green fruit; fold change) was compared with the ratio of Pg‐3‐MG/Pg‐3‐glc, which indicates malonylation activity (Figure S7). Five transcript sequences with correlation coefficients of more than 0.75 were detected. Verification of the sequences using the Genome Database for Rosaceae (GDR, www.rosaceae.org) revealed that some of them consist of more than one *MAT* sequence, for example, maker.Fvb6.2.snap.gene.312.68.mRNA.1 and maker.Fvb6.1.snap.gene.2.58.mRNA.1 (Figure S8A,B). Therefore, the predicted coding regions were used for BLAST searches, with the results allowing the grouping (MAT1 to 9) of sequences based on their similarity (Table S3). Based on the above transcripts, we designed primers, cloned, and sequenced the corresponding genes. The longest open reading frames (ORF) were considered as coding sequences (CDS). Finally, 24 genes from *F. × ananassa* “Senga Sengana” and “Candonga” were cloned, 10 of which could be heterologously translated into protein, of which eight proteins showed enzyme activity. In addition to the previously isolated MAT1C (OR636097), MAT1S (OR636099), and MAT4C_1_ (OR636098), MAT2C_1_ (OR636093), MAT2S_1_ (OR636094), MAT2C_2_ (OR636095), MAT2S_2_ (OR636096), and MAT6C (OR636092) were newly characterized. The protein sequences of MAT2C_1_ and MAT2S_1_ as well as MAT2C_2_ and MAT2S_2_ were identical, so that only MAT2S_1_ and MAT2S_2_ were used for further characterization of the enzymes. All MATs examined have the HXXXD and DFGWG motif, which assigns them to the BAHD acyltransferase family (Figure S9). They also contain the YFGNC motif, characterizing them as anthocyanin acyl co‐transferases. Phylogenetic analysis separates the six different MATs into two groups (MAT6C (OR636092), MAT2S_1_ (OR636094), MAT2S_2_ (OR636096) versus MAT1C (OR636097), MAT1S (OR636099), and MAT4C_1_ (OR636098)), indicating different functions (Figure S9). An analysis of MAT proteins from *F. × ananassa* with known transferases from plants revealed that they are phylogenetically closely related to a malonyl‐CoA: anthocyanidin 5‐O‐glucoside‐6″‐O‐MAT from *Arabidopsis thaliana* (NP_189600.1) and malonyl‐CoA:(iso)flavonoid MAT from *Glycine max* and *Medicago truncatula* (Figure S10) (D'Auria et al., 2007; Dhaubhadel et al., 2008; Suzuki et al., 2007).

### The three additionally identified MAT enzymes only show activity toward flavonol glucosides

The proteins encoded by the additional MAT genes identified by transcriptome analysis in *F. × ananassa* were heterologously produced in *E. coli* and subjected to enzymatic assays. MAT2S_1_ (OR636094), MAT2S_2_ (OR636096), and MAT6C (OR636092) malonylated only K‐3‐glc and Q‐3‐glc, which was verified by LC–MS. MAT6C showed the lowest activity. To better compare the catalytic efficiency of the functional MAT proteins, kinetic studies were performed in which the Michaelis–Menten constant *K*_m_ and the catalytic constant *k*_cat_ were determined for the donor substrate malonyl‐CoA (Table 1) and the acceptor substrates Q‐3‐glc, K‐3‐glc, Pg‐3‐glc, and Cy‐3‐glc (Table 2). The kinetic parameters for MAT6C could not be determined due to the low catalytic activity of the protein. MAT2C_1_/S_1_ and MAT2C_2_/S_2_ showed the lowest *K*_m_ and highest *k*_cat_ values for the donor substrate malonyl‐CoA with the acceptor substrates Q‐3‐glc and K‐3‐glc compared with MAT1C, MAT1S, and MAT4C_1_/S_1_. This results in a catalytic efficiency *k*_cat_/*K*_m_ of 83–138 mm ^−1^ s^−1^ for MAT2C_1_/S_1_ and MAT2C_2_/S_2_ (Table 1). Similarly, the *k*_cat_/*K*_m_ values of MAT2C_1_/S_1_ and MAT2C_2_/S_2_ for the acceptor substrates Q‐3‐glc and K‐3‐glc in the presence of the acceptor malonyl‐CoA were significantly higher (2.9–35 mm ^−1^ s^−1^) than the values for MAT1C, MAT1S, and MAT4C_1_/S_1_ (0.14–0.73 mm ^−1^ s^−1^) (Table 2). Thus, MAT2C_1_/S_1_ and MAT2C_2_/S_2_ exhibit a clear preference for Q‐3‐glc and K‐3‐glc as acceptor substrates. Although comparatively low *k*_cat_/*K*_m_ values for Q‐3‐glc and K‐3‐glc of 0.14–0.68 mm ^−1^ s^−1^ were determined for MAT1C, MAT1S, and MAT4C1/S1, these enzymes are superior to the former MATs as they can malonylate anthocyanidin glucosides. For Pg‐3‐glc and Cy‐3‐glc, MAT1C, MAT1S, and MAT4C1/S1 exhibited *k*_cat_/*K*_m_ values of 0.025–0.25 mm ^−1^ s^−1^.

**Table 1:** Kinetic data of selected MAT enzymes for the donor substrate. s.t.s. signal too small to calculate

Donor	Enzymes	*K*_m_ (mm)	*k*_cat_ (s^−1^)	*k*_cat_/*K*_m_ (mm ^−1^ s^−1^)	Substrate
Malonyl‐CoA	MAT1C	0.70 ± 0.2	0.38 ± 0.06	0.54	Quercetin‐3‐glucoside
	MAT1S	0.45 ± 0.2	0.063 ± 0.009	0.14	Quercetin‐3‐glucoside
	MAT2C_1_/S_1_	0.022 ± 0.01	1.83 ± 0.10	83	Quercetin‐3‐glucoside
		0.020 ± 0.017	2.76 ± 0.3	138	Kaempferol‐3‐glucoside
	MAT2C_2_/S_2_	0.024 ± 0.02	2.39 ± 0.2	100	Quercetin‐3‐glucoside
		0.016 ± 0.02	1.95 ± 0.3	121	Kaempferol‐3‐glucoside
	MAT4C_1_/S_1_	0.41 ± 0.2	0.13 ± 0.02	0.32	Quercetin‐3‐glucoside
	MAT6C	s.t.s.	s.t.s.	s.t.s.	Quercetin‐3‐glucoside
		s.t.s.	s.t.s.	s.t.s.	Kaempferol‐3‐glucoside

**Table 2:** Kinetic data of selected MAT enzymes for acceptor substrates. n.d. not determined, as inactive; s.t.s. signal too small to calculate

Acceptor	Enzyme	*K*_m_ (mm)	*k*_cat_ (s^−1^)	*k*_cat_/*K*_m_ (mm ^−1^ s^−1^)
Quercetin‐3‐glucoside	MAT1C	0.31 ± 0.1	0.21 ± 0.03	0.68
	MAT1S	0.30 ± 0.1	0.060 ± 0.007	0.20
	MAT2C_1_/S_1_	0.18 ± 0.05	4.00 ± 0.2	22
	MAT2C_2_/S_2_	0.18 ± 0.06	6.23 ± 0.3	35
	MAT4C_1_/S_1_	0.22 ± 0.1	0.087 ± 0.01	0.40
	MAT6C	s.t.s.	s.t.s.	s.t.s.
Kaempferol‐3‐glucoside	MAT1C	0.15 ± 0.04	0.11 ± 0.006	0.73
	MAT1S	0.30 ± 0.06	0.043 ± 0.002	0.14
	MAT2C_1_/S_1_	0.79 ± 0.3	4.77 ± 0.6	6.0
	MAT2C_2_/S_2_	1.05 ± 0.4	3.08 ± 0.5	2.9
	MAT4C_1_/S_1_	0.25 ± 0.08	0.072 ± 0.006	0.29
	MAT6C	s.t.s.	s.t.s.	s.t.s.
Pelargonidin‐3‐glucoside	MAT1C	0.27 ± 0.1	0.068 ± 0.01	0.25
	MAT1S	0.40 ± 0.2	0.025 ± 0.006	0.062
	MAT2C_1_/S_1_	n.d.	n.d.	n.d.
	MAT2C_2_/S_2_	n.d.	n.d.	n.d.
	MAT4C_1_/S_1_	0.39 ± 0.2	0.031 ± 0.007	0.080
	MAT6C	n.d.	n.d.	n.d.
Cyanidin‐3‐glucoside	MAT1C	0.017 ± 0.003	0.001 ± 0.0001	0.059
	MAT1S	0.011 ± 0.003	0.0003 ± 0.0001	0.027
	MAT2C_1_/S_1_	n.d.	n.d.	n.d.
	MAT2C_2_/S_2_	n.d.	n.d.	n.d.
	MAT4C_1_/S_1_	0.063 ± 0.03	0.003 ± 0.001	0.048
	MAT6C	n.d.	n.d.	n.d.

### The functional MAT genes are more strongly expressed in *F. × ananassa* “Senga sengana” than in *F. × ananassa* “Candonga” as demonstarted by qPCR

To identify the *MAT* genes responsible for the different concentrations of malonylated anthocyanidin glucosides in the *F. ananassa* cultivars studied, the expression patterns of functional *MAT* genes were determined by quantitative real‐time PCR in fruit, flowers, leaves, and roots of “Candonga” and “Senga Sengana.”

**Figure 5:**
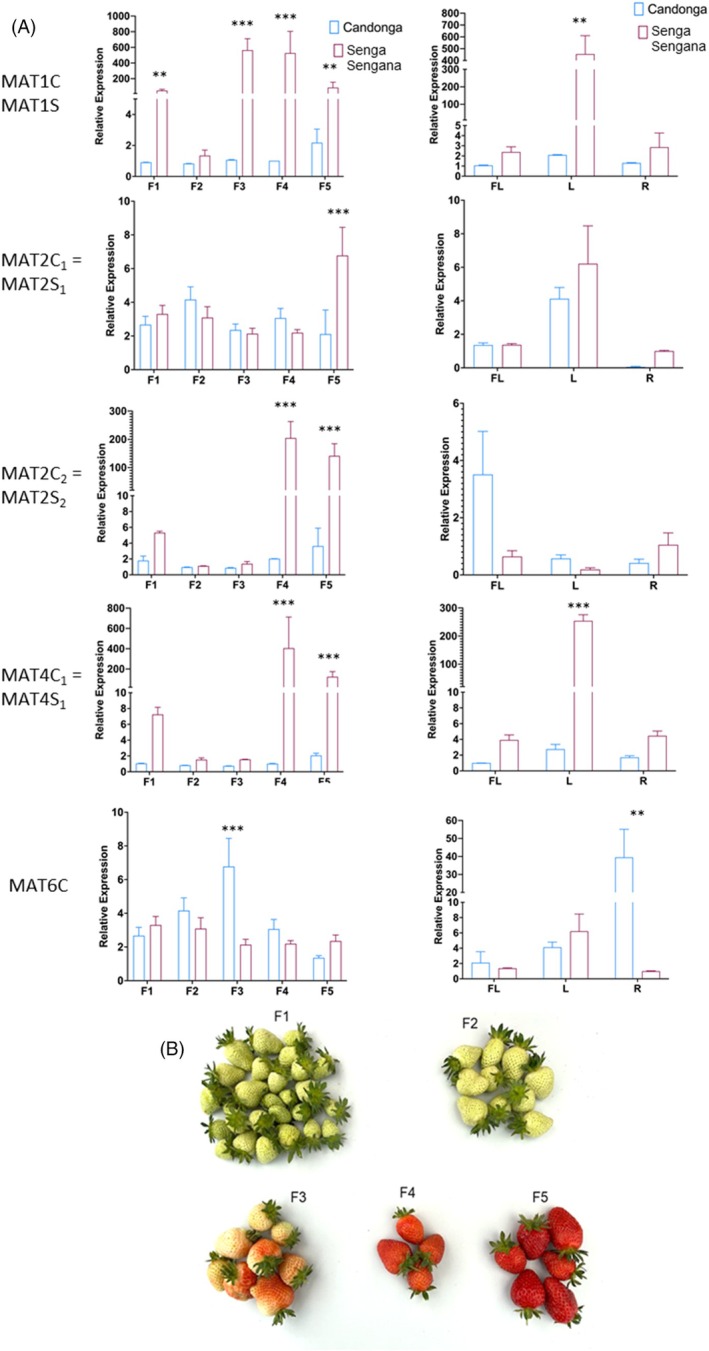
Gene expression analysis by qPCR of MAT genes encoding catalytically active enzymes. (A) The relative quantification of gene expression levels was calculated by comparing the levels of expression of selected MAT genes from two cultivars (“Candonga” and “Senga Sengana”) with the levels of expression of an internal control gene (housekeeping gene FaDBP; XM_004291635.2), which was set to 1. (B) F1 to F5 describe different ripening stages of the fruit while FL, L, and R denote flower, leaf, and root. Statistical significance between samples was determined using Student's *t*‐test (***P < 0.001; **P < 0.01; *P < 0.05). Error bars indicate ± standard errors.

The fruit were picked at five ripening periods from small green (F1) to dark red (F5) to analyze the temporal expression of *MATs* in the fruit (Figure 5). Using qPCR, three biological replicates were measured in each case, and the data were analyzed using the 2^−▵▵Ct^ method. While MAT2C_1_/MAT2S_1_ and MAT6C/MAT6S were expressed at similarly low levels in the fruit of both cultivars, MAT1S, MAT2S_2_, and MAT4S_1_ were approximately 100× more highly expressed in “Senga Sengana” fruit, primarily in the late stages of ripening, than their orthologous genes in the “Candonga” cultivar. The different transcript levels of the latter genes in the two cultivars may therefore be responsible for the different amounts of malonylated anthocyanidin glucosides in the strawberry genotypes.

## DISCUSSION

The aim of the GoodBerry project was to answer the question of how strongly the genotype and environmental factors affect the quality and quantity of value‐determining metabolites in ripe strawberry fruit. For this purpose, the progeny of a cross between *F. × ananassa* “Candonga” and “Senga Sengana” were grown at five different locations in Europe, and the non‐volatile metabolites were analyzed during two harvest years. The genotype of the respective progeny was found to be a significant factor in metabolite differentiation, while cultivation in different climatic zones led to increased variation in metabolite concentrations (Figure 1). The dominant influence of genotype versus growing conditions on the content of secondary metabolites was also found in an association study based on genotypic and environmental data in which *Cyclocarya paliurus* subgroups were identified that contained high levels of bioactive triterpenes (Sun et al., 2022). The content of allergenic proteins in fruit seems to be genotype‐driven as well (Kurze et al., 2018). In the case of the strawberry cross‐population, the flavanols and especially the malonylated anthocyanidin glucosides showed a strong genotype dependency, which was reflected in the parental lines and confirmed results of previous studies (Carbone et al., 2009; Urrutia et al., 2016). For the trait malonylated metabolites, this resulted in a 1:1 split in the progeny for genotypes with less than 200 ppm equ. IS Pg‐3‐MG compared with genotypes with more than 200 ppm equ. IS Pg‐3‐MG (Figure 2). Detailed analyses indicated that the “Senga Sengana” group preferentially malonylates Pg‐3‐glc over Cy‐3‐glc (Figure 3). Since only the sequence of the *F. vesca* genome (v1.0 hybrid) was available at the time of the initial search for candidate genes, available transcriptome data (Härtl et al., 2017) were used to search for MAT genes expressed in ripe fruit (Figure S3). Five identified candidate genes yielded three functionally active promiscuous MAT enzymes that malonylated both flavonoid glucosides and anthocyanidin glucosides (Figure 4) comparable to NbMat1 from *Nicotiana benthamiana* (Liu et al., 2017) and CtMaT1 from *Cistanche tubulosa* (Liu et al., 2024). However, since the gene encoding the most active MAT (MAT4) could only be isolated from the “Candonga,” it was suspected that there are other anthocyanidin‐glucoside MATs in the genome of *F. × ananassa* “Senga Sengana.” A second search for MAT candidate genes was started with the genome of *F. × ananassa* Camarosa (www.rosaceae.org) and a transcriptome dataset of selected progeny of the cross‐population. An *in‐silico* analysis yielded five transcripts whose expression correlated strongly with the content of malonylated anthocyanidin glucosides (correlation coefficient 0.77–0.91) and which occurred 2.7–11.5 times more frequently in “Senga Sengana” than in “Candonga,” as determined by RNA‐seq (Figure S7). The transcripts contained up to four ORFs (Figure S8), with the help of which a further seven functional MAT genes could be isolated through alignments and comparison with the MAT already isolated (Table S3). Four of the isolated MAT genes had an identical sequence in “Candonga” and “Senga Sengana,” including MAT4, which could initially only be isolated from “Candonga” and encodes the most active anthocyanidin‐glucoside MAT. Biochemical analyses showed that the newly characterized MAT2C1/S1, MAT2C2/S2, and MAT6C/6S exclusively malonylate flavonoid glucosides but not anthocyanidin glucosides, whereas MAT1C, MAT1S, and MAT4C_1_/S_1_ transform members of both substrate groups similarly well (Table 2). The different catalytic activities of the characterized MAT are also reflected in the grouping of protein (Figure S10) and gene sequences (Figure S11). The MATs from *F. × ananassa* are more similar to the (iso)flavonoid–glucoside MATs from *G. max* and *M. truncatula* than to the anthocyanidin‐glucoside MATs from other plant species (Figure S10). Targeted quantitative PCR analyses finally provided the answer to the question of which MAT genes are responsible for the different levels of malonylated anthocyanidin glucosides in the two cultivars investigated (Figure 5). Both MAT1S and MAT4S_1_ are expressed about 100 times stronger in “Senga Sengana” compared with their Candonga orthologs, and the encoded proteins show similar levels of anthocyanidin‐glucoside MAT activity (Table 2). The gene product of *MAT2S*_*2*_, which is also highly transcribed in “Senga Sengana,” is likely to be involved in the formation of malonylated flavonoid glucosides. The relative transcript abundance of *MAT* candidate genes was also determined in the fruit of *F. vesca* Ruegen and Yellow wonder (Davik et al., 2020). A strong increase in transcription between the turning and ripe maturity stages was determined for the gene FvH4_6g46743 (in the present study MAT1), while the transcript levels for FvH4_6g46750 (in the present study MAT2, presumably MAT2C_1_) decreased in both cultivars. Thus, the expression pattern of the MAT genes of the *F. vesca* cultivars is similar to that of the corresponding genes from *F. × ananassa* “Candonga” (Figure 5). The present study describes for the first time the characterization and enzymatic activity of 10 MAT enzymes from *F. × ananassa* “Candonga” and “Senga Sengana.” While four *MAT* genes are transcribed in a ripening‐correlated manner, the transcript levels of the other six genes change only slightly, resulting in the absence of an increased formation of malonylated anthocyanidin glucosides in the fruit of “Candonga” during ripening. The formation of malonylated metabolites is thus regulated transcriptionally, recognizable by the fact that the expression of *MAT1* and *MAT4* in “Candonga” is not increased during ripening. We hypothesize that similar to the ripening‐induced *O*‐methyltransferase from *F. × ananassa* (FaOMT) (Zorrilla‐Fontanesi et al., 2012), mutations in *MAT* promoter regions prevent the ripening‐induced expression of functionally active *MAT1* and *MAT4* in different cultivars such as “Candonga” and are inherited.

## CONCLUSION

Given the well‐documented substrate promiscuity of plant malonyltransferases (Liu et al., 2024; Manjasetty et al., 2012; Suzuki et al., 2001; Suzuki et al., 2007), future research should further investigate the activity of FaMATs toward a broader range of acyl donors and flavonoid glucosides. This could unlock new opportunities for industrial applications, particularly in the development of functional foods and nutraceuticals. In parallel, the *in vivo* impact of FaMAT expression on the plant's secondary metabolism remains an important area of study (Chen et al., 2023; Martínez‐Rivas et al., 2023). Understanding how FaMATs influence the biosynthesis of secondary metabolites could provide valuable insights for nutrition science and food chemistry. Moreover, recent findings suggest that plant malonyltransferases play critical roles in responses to abiotic stresses, such as heat, drought, low nitrogen, and alkaline conditions (Ahmad et al., 2017; Trush et al., 2024; Zhang et al., 2025). These insights highlight the potential of malonyltransferases as targets in molecular breeding for stress‐resilient crops. Finally, structural biology and protein engineering of FaMATs offer promising avenues for expanding or tailoring their substrate specificity. Such efforts could facilitate the derivatization of natural products and support the development of plant‐based pharmaceuticals.

## MATERIALS AND METHODS

### Plant material and chemicals

The cultivated strawberries *F. × ananassa* “Candonga” and “Senga Sengana,” in various degrees of ripeness, were supplied by Hansabred GmbH & Co KG. All plant materials were stored at −80°C until use. Micocombichem (Wiesbaden, Germany) and Carbosynth (Berlin, Germany) kindly provided malonyl‐CoA and kaempferol‐3‐glucoside. Quercetin‐3‐glucoside, cyanidin‐3‐glucoside, and pelargonidin‐3‐glucoside were purchased from Extrasynthese (Lyon, France), Roth (Karlsruhe, Germany), and Carbosynth (Berlin, Germany). Other chemicals were purchased from Sigma‐Aldrich (Taufkirchen, Germany), Carl Roth (Karlsruhe, Germany), Fisher Scientific (Schwerte, Germany), and VWR International (Darmstadt, Germany) unless otherwise stated.

### GoodBerry strawberry crossbreeding population

Hansabred GmbH & Co. KG, Dresden, Germany, produced a crossing population of the *F. × ananassa* cultivars “Candonga” and “Senga Sengana.” The resulting F1 generation, as well as the parental lines, were grown at five different locations in Europe (Germany, Poland, France, Spain, and Italy) for 2 years (Table S1). Ten plants were cultivated per genotype and per location. Due to propagation problems, only 18 genotypes bore enough fruit for analysis in the first year of cultivation (Figure S1). In 2028, 64 genotypes produced sufficient fruit. There were isolated losses of individual genotypes at individual locations. After harvesting and removal of the calyx, the ripe fruit were shock‐frozen in liquid nitrogen and stored at −80°C until further processing. As the yield of the different genotypes was unclear, the entire harvested fruit of one genotype was initially combined into a pooled sample. Only after the harvest was complete were the whole or halved fruit roughly homogenized and divided into three biological replicates of equal size. During the crushing process, the strawberries and all equipment used were cooled with liquid nitrogen. The fruit powder was stored at −80°C until analysis. The samples were shipped to the analysis site on dry ice.

### Measurement standards

Some of the data sets took several months to measure. Standards were used for the respective methods to ensure comparable measured values over this longer period. These standards made it possible to check the stability of the measurements and to normalize the results if significant measurement fluctuations occurred between sub‐measurement sets. The starting material for the measurement standards was strawberries purchased from a local supermarket. After removing the calyx, these strawberries were shock‐frozen in liquid nitrogen and ground into a fine powder in batches using a blender under constant cooling with liquid nitrogen. The powder was stored at −80°C until further processing.

### Metabolite profiling of polyphenols

The analysis of the polyphenols was carried out according to a published method (Ring et al., 2013) (Data S1). Five hundred mg of plant material was weighed into a 1.5 ml Eppendorf centrifuge tube. A 0.25 ml methanolic solution of the internal standard Biochanin A (0.2 mg/ml) was added to the plant material. The mixture was briefly homogenized using a vortexer (Vortex‐Genie 2 G‐560E; Scientific Industries, New York, NY, USA). After the addition of 0.25 ml methanol and homogenization (1 min vortexing), the mixture was treated in an ultrasonic bath (Sonorex RK103H; BANDELIN Electronic, Berlin Germany) for 5 min and cooled with ice. The suspension was centrifuged (16,000 *g*, 20 min, 4°C), and the resulting supernatant was transferred to a 2 ml centrifuge tube. The residue was extracted again twice with 0.5 ml methanol each, according to the first extraction. The combined supernatants of the three extractions were concentrated to dryness in a rotary vacuum concentrator (RVC 2‐18; Martin Christ, Osterode am Harz, Germany). The extract was then dissolved with 35 μl MilliQ water. The suspension was vortexed for 1 min and treated in an ultrasonic bath for 10 min. This was followed by centrifugation (16,000 *g*, 20 min, 4°C). The resulting clear supernatant was transferred to a 200 μl insert in a 2 ml amber glass vial for measurement by LC–MS (Methods S1). If the supernatant was cloudy, the centrifugation was repeated. A triplicate determination was performed for each genotype. Fruit samples were analyzed on an Agilent 1100 HPLC/UV system (Agilent Technologies, Waldbronn, Germany) equipped with a reverse‐phase column (Luna 3u C18 100A, 150 × 2 mm; Phenomenex) and connected to a Bruker Esquire3000plus Ion Trap mass spectrometer (Bruker Daltonics, Bremen, Germany). The metabolites were identified using the DataAnalysis program (Bruker Daltonics). Retention time, UV absorption, *m/z* values, and fragmentation patterns were analyzed. The peaks were assigned using an internal database, literature comparison (Aaby et al., 2012; Dias et al., 2015; Gasperotti et al., 2013; Hanhineva et al., 2008; Kårlund et al., 2014; Kårlund et al., 2017; La Barbera et al., 2017; Oszmiański & Wojdyło, 2009), or reference material. Sixty‐three metabolites were quantified in strawberry fruit. The metabolites were quantified via their specific MS trace using the QuantAnalysis program (Bruker Daltonics, Leipzig, Germany). The positive and negative modes were used. In each case, a relative concentration with respect to the internal standard Biochanin A was calculated.

### Gene cloning from DNA

For DNA extraction, 50 mg of homogeneous sample material (fruit or young leaves) was used. The preparation was carried out according to the instructions of the DNeasy® Plant Mini kit (Qiagen, Venlo, The Netherlands). Elution was performed with 100 μl elution buffer for fresh leaf material and with 50 μl for fruit samples. Candidate genes were amplified by PCR (primers in Table S4), and the Nucleo Spin® Gel and PCR Clean‐up kit (Macherey‐Nagel™, Schwerte, Germany) was used to purify the PCR product. The pGEM®‐T Easy kit (Promega, Walldorf, Germany) was used for ligation into the pGEM‐T Vector. In accordance with the manufacturer's recommendation, a molar ratio of fragment to insert of 1:3 was maintained. The ligation was carried out over 3 days at 4°C.

### Gene cloning from RNA and bioinformatics analysis

Total RNA was extracted from *F*. × *ananassa* “Candonga” and “Senga Sengana” using the Qiagen RNeasy Plant Mini Kit for RNA Extraction kit. The first‐strand cDNA was synthesized by Promega M‐MLV Reverse Transcriptase (USA) as a gene cloning template. The coding sequences of all FaMAT genes were amplified using the New England Biolabs Phusion High‐Fidelity DNA Polymerase (Ipswich, MA, USA). Cycling conditions were as follows: 98°C for 30 s followed by 35 cycles of 98°C for 10 s, 60°C for 30 s, and 72°C for 45 s, then a final extension at 72°C for 10 min (SensoQuest labcycler; SensoQuest, Göttingen, Germany). Fragments were ligated to pGEM‐T Vector from Promega. C‐FaMAT primers were used for gene cloning, P‐FaMAT primers for protein expression with Sma I and Not I as restriction sites (Table S4). DNA amplicons were digested and inserted into the pGEX‐4 T‐1 vector after the GST tag. The recombinant plasmid obtained was then transformed into *Escherichia coli* BL21 (DE3) (Novagen, Darmstadt, Germany) and confirmed by sequencing. Alignment of sequences was generated using ClustalW. The neighbor‐joining phylogenetic tree was drawn using MEGA version 10.2 software.

### Protein expression

The expression and purification of the proteins were performed according to Schulenburg et al. (2016). Recombinant strains were inoculated in 1 L LB medium with 0.1 antibiotics, then incubated at 37°C and 150 rpm until OD600 = 0.6. Protein expression was introduced by 1 mol/L IPTG, followed by incubation for 24 h at 18°C and 200 rpm. The cell suspension was collected by 5000 *g*, for 10 min, at 4°C. To purify the recombinant glutathione S‐transferase (GST) fusion proteins, a GST Bind Resin (Novagen) was used. The binding buffer (4.3 mm Na_2_HPO_4_, 1.47 mm KH_2_PO_4_, 137 mm NaCl, 2.7 mm KCl, pH 7.3) contained 100 μm of phenylmethylsulfonyl fluoride as a proteinase inhibitor. After seven elution steps with GST elution buffer (10 mm glutathion reduced, 50 mm Tris/HCl, pH 8.0), the total protein concentration of crude protein, wash fractions, and all elution fractions was quantified via Bradford Assay (Schulenburg et al., 2016). For further activity assays, elution fractions with a total protein concentration c > 450 μg/μl were combined for each protein, respectively. The purity of the recombinant proteins was checked via SDS‐PAGE (Figure S6).

### Malonyltransferase assay

The standard enzymatic assays were performed in a 50 μl mixture containing 0.6 mm malonyl‐CoA as malonyl‐donor, 1.2 mm malonyl‐acceptor, 1 mm EDTA, 10 mm β‐mercaptoethanol, and 2.5 μg purified FaMATs protein in 20 mm potassium phosphate buffer (pH 7.0). The reactions were incubated at 30°C for 12 hr in Thermomixer comfort (Eppendorf, Hamburg, Germany), and terminated by adding twice the volume of ice‐cold methanol. The precipitated proteins were removed by centrifugation at max speed at 4°C for 30 min, and the 50 μl supernatants were subjected to LC–MS (Agilent 1100 Series; Agilent Technologies, Santa Clara, CA, USA coupled with Bruker Daltonics Esquire 3000plus Ion Trap; Bruker Daltonics, Bremen, Germany) analysis (Methods S1). For kinetics studies of FaMATs, the reaction mixture of the malonyltransferase activity assay consisted of the following components: 2.5 μg protein, 2 μl 30 mm substrate in DMSO, 5 μl potassium phosphate buffer (pH 7.0), 3 μl malonyl‐CoA (10 mm in MQ water), fill up to 50 μl with MQ water. After homogenization, the mixture was incubated for 30 min at 400 rpm and 30°C in Thermomixer comfort (Eppendorf). The reaction was stopped by adding 100 μl methanol. This was followed by centrifugation (10 min, 4°C, 16000 *g*). For analysis by LC–MS, 50 μl of the supernatant was pipetted into an insert/vial. The kinetic parameters, including the Michaelis–Menten constant (*K*_m_) and *v*_max_, were calculated by non‐linear regression analysis using GraphPad Prism 7 (GraphPad Software, Boston, MA, USA).

### RNA‐seq analysis

Expression analysis by RNA‐seq of the fruits was performed using three biological replicates according to (Sánchez‐Sevilla et al., 2017). For each biological replicate, a paired‐end library with an approximate insert size of 300 bp was prepared using an optimized Illumina protocol at the Centro Nacional de Análisis Genómicos (CNAG, Barcelona, Spain). The libraries were sequenced on an Illumina HiSeq 2000 with 2 × 100 bp reads. More than 30 million reads were generated for each sample. The fruit of genotypes H15, H16, H25, H32, H36, H37, H40, H48, H50, H51, H53, H54, H58, H61, H67, H69, H71, H73, H76, H83, H87, H94, H97, H98, H103, H110, H112, H114, H120, H122, H125, H126 were subjected to RNA‐seq analysis.

### Quantitative real‐time PCR

The cDNA synthesis for qPCR used the iScript cDNA Synthesis Kit from BIO‐RAD (Hercules, CA, USA). Specific qPCR primers were designed to amplify 198 bp products from all FaMATs, respectively, and the specificity of each primer pair was confirmed by BLAST in NCBI, PCR product sequencing, and detection of a single peak of fluorescence from melt curves during qPCR (Table S4). cDNA was diluted to 50 ng/μl in sterile RNase‐free water. Each sample had a reaction volume of 20 μl, consisting of 2 μl of diluted cDNA and 400 nmol/L of each primer in SensiFAST SYBR Hi‐ROX Mix from Meridian Bioscience (Cincinnati, OH, USA). Cycling conditions were as follows: initial activation at 95°C for 2 min followed by 40 cycles of 95°C for 5 s, 60°C for 10 s, and 72°C for 20 s. Reactions were then heated to 95°C for 5 min, cooled to 50°C for 5 s, then heated to 95°C to produce melt curves. Normalization was performed against an average value obtained from the housekeeping gene FaDBP (XM_004291635.2) and is expressed as relative transcript levels. A triplicate analysis was conducted for each sample.

## AUTHOR CONTRIBUTIONS

WGS, TH, KO, SO, and BD designed the experimental plan. XW, JT, MW, AH, ZY, XZ, WS, CL, CY, TDH, and HH conducted most of the experiments. KO, SO, IA, JFSS, BD, AP, PC, LM, BM, and AM provided the strawberry samples. BU and FZ carried out the bioinformatic analyses. WGS, JT, TH, TDH, and XW wrote the manuscript.

## CONFLICT OF INTEREST

The authors declare no conflicts of interest.

## Supporting information


**Table S1.** Locations of the GoodBerry crossing population.
**Table S2.** Metabolites analysed by LC‐MS.
**Table S3.** Gene assignments. Malonyltransferasegenes from *Fragaria*sp. in GDR, Genome Database for Rosaceae(www.rosaceae.org); NCBI, National Centerfor Biotechnology Information (www.ncbi.nlm.nih.gov), and isolated from ‘Candonga’ (C) and from ‘Senga Sengana’ (S).
**Table S4.** Primer sequences for gene cloning, protein expression and quantitative PCR.
**Figure S1.** Generation of the cross population, number of progeny, number of genotypes analysed and genotypes analysed per year.
**Figure S2.** Formation of malonylated pelargonidin‐3‐glucoside facilitates the accumulation of larger amounts of pelargonidin metabolites.
**Figure S3.** Heat map of the transcript levels of *MAT* candidate genes in *Fragaria vesca* cultivars.
**Figure S4.** Agarose gel of the amplified MAT candidate genes.
**Figure S5.** *FaMAT* candidates from *F. xananassa* ‘Candonga’ and ‘Senga Sengana’ selected based on a transcriptome analysis of *F. vesca* varieties.
**Figure S6.** SDS‐PAGE analysis of recombinant MAT proteins.
**Figure S7.** Correlation analysis of transcripts and the ratio of Pg‐3‐MG and Pg‐3‐glc.
**Figure S8.** Excerpts from the Genome Database for Rosaceae (www.rosaceae.org).
**Figure S9.** Protein sequence analysis of enzymatically active MAT enzymes.
**Figure S10.** Phylogenetic analysis of the protein sequences of functional MAT enzymes from different plant species.
**Figure S11.** Phylogenetic analysis of the nucleotide sequences of selected MAT genes from different Fragaria species.
**Methods S1.** LC‐MS system.

**Data S1.** Metabolites GoodBerry.

## Data Availability

All relevant data can be found within the manuscript and the supporting information.
