# Supplementary material for: Inheritance and enzymatic basis of anthocyanin malonylation in Fragaria × ananassa
Source: Plant J. 2025 Oct 24;124(2):e70485. doi: 10.1111/tpj.70485 (PMC12551991; doi:10.1111/tpj.70485)
Supplement: Supplementary file 1 — Table S1. Locations of the GoodBerry crossing population. Table S2. Metabolites analysed by LC‐MS. Table S3. Gene assignments. Malonyltransferasegenes from Fragariasp. in GDR, Genome Database for Rosaceae(www.rosaceae.org); NCBI, National Centerfor Biotechnology Information (www.ncbi.nlm.nih.gov), and isolated from ‘Candonga’ (C) and from ‘Senga Sengana’ (S). Table S4. Primer sequences for gene cloning, protein expression and quantitative PCR. Figure S1. Generation of the cross population, number of progeny, number of genotypes analysed and genotypes analysed per year. Figure S2. Formation of malonylated pelargonidin‐3‐glucoside facilitates the accumulation of larger amounts of pelargonidin metabolites. Figure S3. Heat map of the transcript levels of MAT candidate genes in Fragaria vesca cultivars. Figure S4. Agarose gel of the amplified MAT candidate genes. Figure S5. FaMAT candidates from F. xananassa ‘Candonga’ and ‘Senga Sengana’ selected based on a transcriptome analysis of F. vesca varieties. Figure S6. SDS‐PAGE analysis of recombinant MAT proteins. Figure S7. Correlation analysis of transcripts and the ratio of Pg‐3‐MG and Pg‐3‐glc. Figure S8. Excerpts from the Genome Database for Rosaceae (www.rosaceae.org). Figure S9. Protein sequence analysis of enzymatically active MAT enzymes. Figure S10. Phylogenetic analysis of the protein sequences of functional MAT enzymes from different plant species. Figure S11. Phylogenetic analysis of the nucleotide sequences of selected MAT genes from different Fragaria species. Methods S1. LC‐MS system. [file TPJ-124-0-s002.pdf]

Supplemental information

Inheritance and enzymatic basis of anthocyanin malonylation in *Fragaria × ananassa*

Xiran Wang [1], Johanna Trinkl [1], Martha Wulanjati [1], Annika Haugeneder [1], Klaus Olbricht [2], Sonia Osorio [3], Iraida Amaya [4], José F. Sánchez-Sevilla [5], Béatrice Denoyes [6], Aurélie Petit [7], Philippe Chartier [7], Luca Mazzoni [8], Bruno Mezzetti [8], Agnieszka Masny [9], Björn Usadel [10], Freya Ziegler [10], Zhen Yu [1], Xiaotong Zhu [1], Wenhao Shi [1], Chan Li [1], Chiye Yuan [1], Huiyun Hu [1], Timothy D. Hoffmann [1], Thomas Hoffmann [1], Wilfried G. Schwab [1]

[1] Biotechnology of Natural Products, School of Life Sciences, Technische Universität München, Liesel-Beckmann-Str. 1, 85354 Freising, Germany
[2] Hansabred GmbH & Co. KG, Radeburger Landstr. 12, 01108 Dresden, Germany
[3] Departamento de Biología Molecular y Bioquímica, Instituto de Hortofruticultura Subtropical y Mediterránea "La Mayora", Universidad de Málaga, Consejo Superior de Investigaciones Científicas, Campus de Teatinos, 29071 Málaga, Spain
[4] Plant Breeding and Physiology, Instituto de Hortofruticultura Subtropical y Mediterránea "La Mayora", Universidad de Málaga, Consejo Superior de Investigaciones Científicas, Campus de Teatinos, 29010 Málaga, Spain
[5] Centro IFAPA de Málaga, Instituto Andaluz de Investigación y Formación Agraria y Pesquera (IFAPA), 29140, Málaga, Spain
[6] INRAE, Biologie du Fruit et Pathologie, UMR 1332, INRAE, Université Bordeaux, F-33140 Villenave d'Ornon, France
[7] INVENIO, 33800, Bordeaux, France
[8] Dipartimento di Scienze Agrarie, Alimentari e Ambientali, Università Politecnica delle Marche, 60131, Ancona, Italy
[9] Department of Horticultural Crop Breeding, The National Institute of Horticultural Research, Konstytucji 3 Maja 1/3, 96-100, Skierniewice, Poland
[10] Institute of Bio- and Geosciences, Bioinformatics (IBG-4), Forschungszentrum Jülich GmbH, 52428, Jülich, Germany

Supplementary Tables

Table S1: Locations of the GoodBerry crossing population

Projekt partner	Hansabred	INHORT	Ciref	IFAPA	UPM
**country**	Germany	Poland	France	Spain	Italy
**location**	51°8′42″N	51°95’N	44°85’N	37º12’22’’N	43°31′ N
**soil type**	sandy loam on gravel soil	Pseudo-podzolic soil with light loam	soilless	sandy	40% clay, 25% sand and 35% silt; 9% active lime
**soil pH**	4.9	6.5-7.0	6.8	6.5-7.0	7.9
**cultivation conditions**	open field	open field	soilless, under tunnel	Plastic tunnel, plastic mulch, earth dam	plastic mulch, earth dam
**Watering**	irrigation	Drip irrigation	Drip irrigation	Drip irrigation	Drip irrigation
**Protection against frost damage**	depending on the weather, an anti-frost fleece	no	no	from mid november	no

Table S2: Metabolites analysed by LC-MS

RT [min]	Metabolit	+*m/z*	-*m/z*	+MS2	-MS2
2.7	Malic acid		133 [M-H]$^-$		115
3.5	Ascorbic acid		175 [M-H]$^-$		115
4.0	Glutathione red.	308 [M+H]$^+$	306 [M-H]$^-$	179, 162, 233, 116	254, 272, 288, 179
4.8	Citric acid		191 [M-H]$^-$		191, 111, 173, 129
6.4	Chelidonic acid		183 [M-H]$^-$		183, 139, 111, 95
9.5	Glutathione ox.	613 [M+H]$^+$	611 [M-H]$^-$	484, 355	306, 482, 338
10.8	Phenylalanine	166 [M+H]$^+$	164 [M-H]$^-$	120	
13.2	(Epi)catechin-(epi)catechin-(epi)catechin isomer 1		865 [M-H]$^-$		695, 577, 575, 739, 847, 287
14.4	(Epi)afzelechin-(epi)catechin-(epi)catechin		849 [M-H]$^-$		577, 559, 287
16.0	(Epi)catechin hexoside		451 [M-H]$^-$		289, 245, 161
16.0	HDMF-Glc (agylcon trace)	129 [M-Glc+H]$^+$			
16.0	HDMF-Glc (Glc trace)	313 [M+Na]$^+$		185, 151, 285	
16.6	Tryptophane	205 [M+H]$^+$	203 [M-H]$^-$	188	
17.3	Galloyl-HHDP glucose		633 [M-H]$^-$		633, 301, 481, 275
17.3	Procyanidin B3		577 [M-H]$^-$		425, 559, 451, 407, 287, 289
18.0	Procyanidin B3+B1		577 [M-H]$^-$		425, 407, 451, 559, 287
18.4	(Epi)catechin-(epi)catechin-(epi)catechin		865 [M-H]$^-$		425, 449, 451, 407, 289, 245
18.7	(Epi)catechin-(epi)catechin-(epi)catechin isomer 2		865 [M-H]$^-$		695, 577, 575, 739, 847, 287
18.9	Procyanidin B1		577 [M-H]$^-$		425, 559, 451, 407, 287, 289
19.4	(Epi)catechin-(epi)catechin-(epi)catechin isomer 3		865 [M-H]$^-$		695, 577, 575, 739, 847, 287
19.7	HHDP-galloyl glucosid		633 [M-H]$^-$		301, 463
20.5	(Epi)afzelechin-(epi)catechin isomer 1		561 [M-H]$^-$		289, 543, 407, 435,271, 245, 164
20.5	Catechine	291 [M+H]$^+$	289 [M-H]$^-$	123, 139, 165, 273	245
21.0	Digalloyl-quinic acid		495 [M-H]$^-$		343, 325, 191, 169
21.1	Caffeic acid-glucose ester/-glucoside		341 [M-H]$^-$		179 161 203
21.5	(Epi)catechin-(epi)catechin isomer		577 [M-H]$^-$		425, 407, 451, 559, 289
21.6	Epiafzelechin-(4a→8)-pelargonidin-3-*O*-glucoside	705 [M]$^+$		543, 525, 407, 313	
22.0	(Epi)afzelechin-(epi)afzelechin-(epi)catechin isomer 1		833 [M-H]$^-$		543, 561, 707, 815, 679, 469, 435, 417, 289, 271
22.0	Digalloyl-hexose		483 [M-H]$^-$		271, 467, 331, 313, 211, 169
22.5	Cyanidin-3-glucoside	449 [M]$^+$		287	
22.8	p-Cumaryl-glucose ester		325 [M-H]$^-$		325, 163, 119
23.4	Digalloyl-HHDP-glucose		785 [M-H]$^-$		615, 301
23.7	Galloyl-bis-HHDP-glucose isomer		935 [M-H]$^-$		633, 451, 301
23.8	(Epi)afzelechin-(epi)afzelechin-(epi)catechin isomer 2		833 [M-H]$^-$		543, 561, 707, 815, 679, 407, 469, 435, 417, 289, 271, 790
24.0	Ferulic acid-glucose ester/-glucoside		355 [M-H]$^-$		193, 217, 175
24.1	(Epi)afzelechin-(epi)catechin isomer 2		561 [M-H]$^-$		289, 543, 407, 435,271, 245, 164
24.3	Pelargonidin-3-glucoside	433 [M]$^+$		271	
24.8	Pelargonidin-3-rutinoside	579 [M]$^+$		271, 433	
25.1	Trisgalloyl-glucose/hexose		635 [M-H]$^-$		465
25.9	Ellagic tannin		937 [M-H]$^-$		767, 301, 993, 841, 785, 741, 713, 635, 617, 489, 571, 553, 483, 465, 419, 313, 275
27.3	Kaempferol-hexose-glucuronide		623 [M-H]$^-$		461, 447, 285, 327, 503, 605
27.7	Tetragalloyl-glucose/hexose		787 [M-H]$^-$		617, 635, 573, 465
29.0	(Epi)afzelechin-(epi)afzelechin-(epi)catechin isomer 3		833 [M-H]$^-$		543, 561, 707, 815, 679, 469, 435, 417, 289, 271
29.1	Cyanidin-3-malonylglucoside	535 [M]$^+$		287	
29.5	Pelargonidin-3-malonylglucoside	519 [M]$^+$		475,433, 271	
30.2	Cinnamic acid-glucose ester	333 [M+Na]$^+$	355 [M+HCOO]$^-$	185	309, 207, 147
31.6	Quercetin-pentose-glucuronide	611 [M+H]$^+$	609 [M+H]$^-$	479, 303	301, 177
32.3	Kaempferol-dihexoside	625 [M+H]$^+$	623 [M-H]$^-$	463, 287	285, 337
33.6	Quercetin-3-pentoside	435 [M+H]$^+$	433 [M-H]$^-$	303	301
33.7	Quercetin-3-glucoside	465 [M+H]$^+$	463 [M-H]$^-$	303	301
34.1	Kaempferol-pentose-glucuronide	595 [M+H]$^+$	593 [M+H]$^-$		285, 307, 429
34.4	Quercetin-3-glucuronide	479 [M+H]$^+$	477 [M-H]$^-$	303	301
34.9	Ellagic acid		301 [M-H]$^-$		301
36.4	Kaempferol-3-glucoside		447 [M-H]$^-$		447, 327, 285, 255
36.5	Kaempferol-glucuronide	463 [M+H]$^+$	461 [M-H]$^-$	287	285
36.6	Quercetin-3-malonylglucoside		505 [M-CO_2-H]$^-$	303	301
36.6	Quercetin-3-acetylglucoside	507 [M+H]$^+$	505 [M-H]$^-$	303	301
37.6	Dicaffeoyl-cumaryl-spermidine	616 [M+H]$^+$	614 [M-H]$^-$	454, 470, 436	
37.9	Caffeoyl-dicoumaryl-spermidine	600 [M+H]$^+$	598 [M-H]$^-$	438, 420	478, 550, 436, 358
38.1	Kaempferol-3-malonylglucoside		489 [M-CO_2-H]$^-$	287	285
38.1	Kaempferol-3-acetyglucoside	491 [M+H]$^+$	489 [M-H]$^-$	287	285
38.6	Kaempferol-3-coumarylglucoside	595 [M+H]$^+$	593 [M-H]$^-$	285, 447, 547	285, 447, 547
38.8	Dicoumaryl-hexose	495 [M+Na]$^+$	471 [M-H]$^-$		307, 427, 163, 453, 325, 291, 163, 187, 145
41.0	Biochanin A (internal standard)	285 [M+H]$^+$	283 [M-H]$^-$	285	283

Table S3: Gene assignments. Malonyltransferase genes from *Fragaria* sp. in GDR, Genome Database for Rosaceae (www.rosaceae.org); NCBI, National Center for Biotechnology Information (www.ncbi.nlm.nih.gov), and isolated from 'Candonga' (C) and from 'Senga Sengana' (S).

**gene (GDR)**	**code (this publication)**	**gene (GDR)**	**gene (NCBI)**	**gene (GDR)**	**gene (NCBI)**
**(F. vesca v1.0 hybrid)**	**(F. x ananassa var. Candonga (C) or Senga Sengana (S)**	**(F. vesca v4.0 a2)**	**(F. vesca subsp. veca; NCBI)**	**F. x ananassa var. Camarosa transcript**	**NCBI Nr.**
4261	MAT1C<br>MAT1S	FvH4_6g46743	XM_011470347	maker-Fvb6-2-snap-gene-312.68-mRNA-1<br>maker-Fvb6-3-snap-gene-38.70-mRNA-1	OR636097 (Candonga)<br>OR636099 (Senga Sengana)
4262	MAT2C$_1$ = MAT2S$_1$<br>MAT2C$_2$ = MAT2S$_2$<br>MAT2S$_3$ (premature stop)	FvH4_6g46750	XM_004306019<br>XM_011469577 (short)	maker-Fvb6-1-snap-gene-2.58-mRNA-1<br>maker-Fvb6-2-snap-gene-312.68-mRNA-1	OR636093 (Candonga) OR636094 (Senga Sengana)<br>OR636095 (Candonga) OR636096 (Senga Sengana)
3835	MAT3C = MAT3S	FvH4_6g48770	XM_004306104	maker-Fvb6-1-augustus-gene-3.44-mRNA-1	
29347	MAT4C$_1$ = MAT4S$_1$<br>MAT4S$_2$ (premature stop)	FvH4_7g01410<br>FvH4_7g01310	XM_004308633<br>XM_011472261	augustus_masked-Fvb7-2-processed-gene-12.2-mRNA-1	OR636098 (Candonga)
4266	MAT5C<br>MAT5S	FvH4_6g46780	XM_011469579	augustus_masked-Fvb6-1-processed-gene-44.15-mRNA-1<br>maker-Fvb6-2-snap-gene-312.67-mRNA-1	
4257	MAT6C = MAT6S	FvH4_6g46740	XM_004306016	maker-Fvb6-2-snap-gene-312.68-mRNA-1<br>maker-Fvb6-1-snap-gene-2.58-mRNA-1	OR636092 (Candonga)<br>OR636091 (Senga Sengana)
4258 (truncated)	MAT7 (truncated MAT2)	FvH4_6g46741<br>(truncated)	XM_011470346<br>(truncated)	maker-Fvb6-1-snap-gene-2.58-mRNA-1	
4259	MAT8 (related with MAT4)	FvH4_6g46742	XM_004304176	maker-Fvb7-4-augustus-gene-211.16-mRNA-1	
4264	MAT9	FvH4_6g46770	XM_011469578	maker-Fvb6-1-snap-gene-2.57-mRNA-1	ON229037 (Senga Sengana)

Table S4: Primer sequences for gene cloning, protein expression and quantitative PCR

**Cloning of candidates from DNA**		
Gene04261_f_untr	5'-CTGGTTCAATCTCTAGTCTGTCTCTCCA-3'	Metabion, Planegg
Gene04261_r_untr	5'-GCAACAACATTAACACCATTATTATTGCT-3'	Metabion, Planegg
Gene04261_f_ATG	5'-ATGGCACATCCAAACTCACTTGTAAATG-3'	Metabion, Planegg
Gene04261_r_ATG	5'-TCAAAGGTTTTCAAGACCTTTGGCAAATTGTG-3'	Metabion, Planegg
Gene04262_f_untr	5'-CATTTTCTATCTCTTTGCTCCTTCGTAAGTA-3'	Metabion, Planegg
Gene04262_r_untr	5'-GTTTCTCTTGGTCCATAATCTCAATC-3'	Metabion, Planegg
Gene04262_f_ATG	5'-ATGGCAAACTTATCAGTGAAGAAAGTTGAGG-3'	Metabion, Planegg
Gene04262_r_ATG	5'-CTAATGCACTAGGCTAGCAAAGAGAGCA-3'	Metabion, Planegg
Gene03835_f_untr	5'-AGTTTCAATCAATTCAGCATCATCTCCA-3'	Metabion, Planegg
Gene03835_r_untr	5'-ATTGGGCCTTGGTGTACATATGGCT-3'	Metabion, Planegg
Gene03835_f_ATG	5'-ATGGCATCTCCAAACTCATCTGCAAGAG-3'	Metabion, Planegg
Gene03835_r_ATG	5'-TCAATTTCCCTTGAGACCTTCGGCAAATAG-3'	Metabion, Planegg
Gene29347_f_untr	5'-CTTCTTCTAATCTTCTTCTTCCCCA-3'	Metabion, Planegg
Gene29347_r_untr	5'-ATAGGGCTTTTCTCAACTCCTCTCAC-3'	Metabion, Planegg
Gene29347_f_ATG	5'-ATGGAGCAACCAAGCTCGGTGAAACTGG-3'	Metabion, Planegg
Gene29347_r_ATG	5'-TCAGTGTTTTCCAAAACCTTTAGCAAATAGAG-3'	Metabion, Planegg
Gene04266_f_untr	5'-CAAGTTTCTTCCAATTGATCATCTCTA-3'	Metabion, Planegg
Gene04266_f_ATG	5'-ATGGCTTTCCGAAACTCAACTACAAAAGTGG-3'	Metabion, Planegg
Gene04266_r_ATG	5'-TCAAGCTGGAGCCGATACCTCCCAGGCGGTTTTTGACCTCGCG-3'	Metabion, Planegg
**Protein expression**		
C1-f	5'-ATGGATCCATGGCACATCCAAACTCACTTG-3'	Metabion, Planegg
C1-r	5'-ATGCGGCCGCTCAAAGGTTTTCAAGGCCTTTG-3'	Metabion, Planegg
C2-f	5'-ATGGATCCATGGCAAACTTATCAGTGAAGAAAG-3'	Metabion, Planegg
C2-r	5'-ATGCGGCCGCCTAATGCACTAGGCTAGCAAAG-3'	Metabion, Planegg
C3-f	5'-ATGGATCCATGGCATCTCCAAACTCATCTG-3'	Metabion, Planegg
C3-r	5'-ATGCGGCCGCTCAGCCTTGACTAAACAAACCC-3'	Metabion, Planegg
C4-f	5'-ATGGATCCATGGAGCAACCAAGCTCGG-3'	Metabion, Planegg
C4-r	5'-ATGCGGCCGCTCAGTGTTTTCCAAAACCTTTAG-3'	Metabion, Planegg
S1-f	5'-ATGGATCCATGGCACATCCAAACTCACTTG-3'	Metabion, Planegg
S1-r	5'-ATGCGGCCGCTCAAAGGTTTTCAAGGCCTTTG-3'	Metabion, Planegg
C5-f	5'-ATGGATCCATGGCTTTCCGAAACTCAACTAC-3'	Metabion, Planegg
C5-r	5'-ATGCGGCCGCTCAAATCCTAGAATGGTTTTTGAG-3'	Metabion, Planegg
**Cloning from cDNA**		
C-MAT1-F	ATGGGCTAATAATAACATTCTAC	Eurofins, Ebersberg
C-MAT1-R	CTACCAATATAATTTCATCTCAAATTTA	Eurofins, Ebersberg
C-MAT2C1S1-F	CGCGGATCCGCGATGGCAAACTTATCAGTG	Eurofins, Ebersberg
C-MAT2C1S1-R	CCGCTCGAGCGGCTAATGCACTAGGC	Eurofins, Ebersberg
C-MAT2C2S2-F	ATGGCAAACTTATCAGTG	Eurofins, Ebersberg
C-MAT2C2S2-R	TTAAAAAAAATATATAAAAAAATATTACCAAACA	Eurofins, Ebersberg
C-MAT4C1S1-F	ATGGAGCAACCAAGCTCGGTGAAACTGG	Eurofins, Ebersberg
C-MAT4C1S1-R	TCAGTGTTTTCCAAAACCTTTAGCAAATAGAG	Eurofins, Ebersberg
C-MAT6C-F	ATGAAGACTGTGGAGTTTGGGGAG	Eurofins, Ebersberg
C-MAT6C-R	CTACATCAATCTTCAATTAAAGACCTCTGGCAAAT	Eurofins, Ebersberg
**Protein expression**		Eurofins, Ebersberg
P-MAT1-F	CCCCGGGGATGGCACATCCAAACTCACTTGTAAAT	Eurofins, Ebersberg
P-MAT1-R	TTGCGGCCGCAATCAAAGGTTTTCAAGGCCTTTGGC	Eurofins, Ebersberg
P-MAT2C1S1-F	CCCCGGGGATGGCAAACTTATCAGTGAAGAAAGTTGAGG	Eurofins, Ebersberg
P-MAT2C1S1-R	TTGCGGCCGCAACTAATGCACTAGGCTAGAAAACAGAGCA	Eurofins, Ebersberg
P-MAT2C2S2-F	CCCCGGGGATGGCAAACTTATCAGTGAAGAAAGTTGAGG	Eurofins, Ebersberg
P-MAT2C2S2-R	TTGCGGCCGCAACTAATGCACTAGGCTAGCAAAGAGAGC	Eurofins, Ebersberg
P-MAT4C1S1-F	ATGGATCCATGGAGCAACCAAGCTCGG	Eurofins, Ebersberg
P-MAT4C1S1-R	ATGCGGCCGCTCAGTGTTTTCCAAAACCTTTAG	Eurofins, Ebersberg
P-MAT6C-F	CCCCGGGGATGGCAAACTCAAACTCAGTGAAAGT	Eurofins, Ebersberg
P-MAT6C-R	TTGCGGCCGCAATTAAAGACCTCTGGCAAATAGAGAAGCG	Eurofins, Ebersberg
**qPCR**		Eurofins, Ebersberg
q-DBP-F	TTGGCAGCGGGACTTTACC	Eurofins, Ebersberg
q-DBP-R	CGGTTGTGTGACGCTGTCAT	Eurofins, Ebersberg
q-MAT1-F	ACTGCATAGCAGGCCGTTTG	Eurofins, Ebersberg
q-MAT1-R	TGTGAGCCAGCAACTGAAGC	Eurofins, Ebersberg
q-MAT2C1S1-F	TTACCAAACCTCTTCCACCG	Eurofins, Ebersberg
q-MAT2C1S1-R	AGGTGGTCGAATCAGGATC	Eurofins, Ebersberg
q-MAT2C2S2-F	CCTAAGCTACGTTCAAGGCG	Eurofins, Ebersberg
q-MAT2C2S2-R	CGATAGAGAAGCCTCGGTTG	Eurofins, Ebersberg
q-MAT4C1S1-F	GGACTGTAGGTCTCGCTTGG	Eurofins, Ebersberg
q-MAT4C1S1-R	GAATCCCTCAACCCCAAAAT	Eurofins, Ebersberg
q-MAT6C-F	GCTCACCGGACTCAATCACA	Eurofins, Ebersberg
q-MAT6C-R	CGGCCAGAGGAAGAAAATGT	Eurofins, Ebersberg

Supplementary Figures

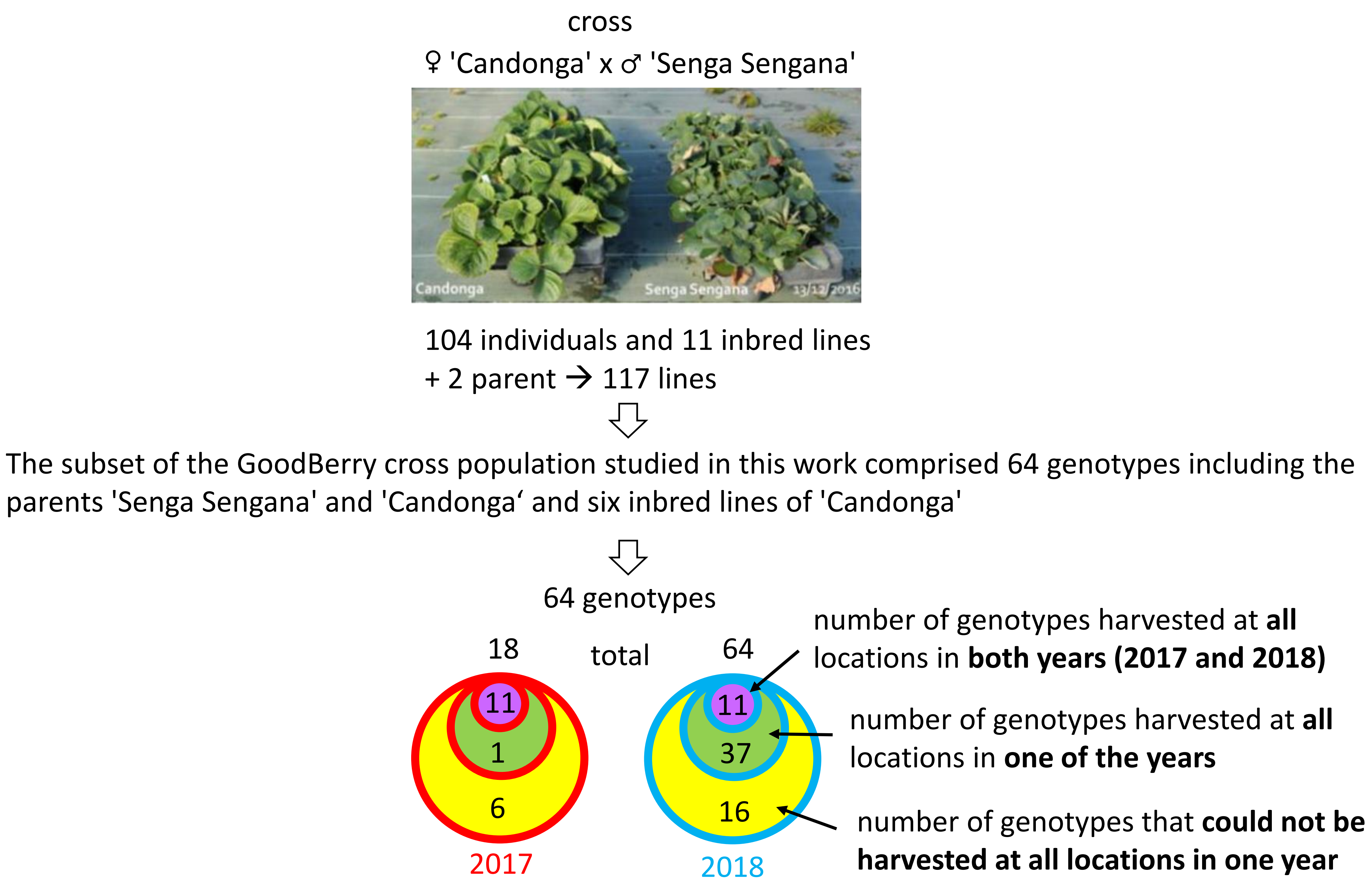

Figure S1. Generation of the cross population, number of progeny, number of genotypes analysed and genotypes analysed per year. A cross population of the *F.* × *ananassa* 'Candonga' and 'Senga Sengana' was produced. The resulting pseudo full-sibling F1 population consisted of 104 individuals and 11 inbred lines. A subset of 64 genotypes was selected for further analysis. In 2017, enough fruits were obtained from 18 genotypes to analyze their metabolites, while in 2018, 48 genotypes produced sufficient fruits for analyses. Eleven genotypes could be examined in both years.

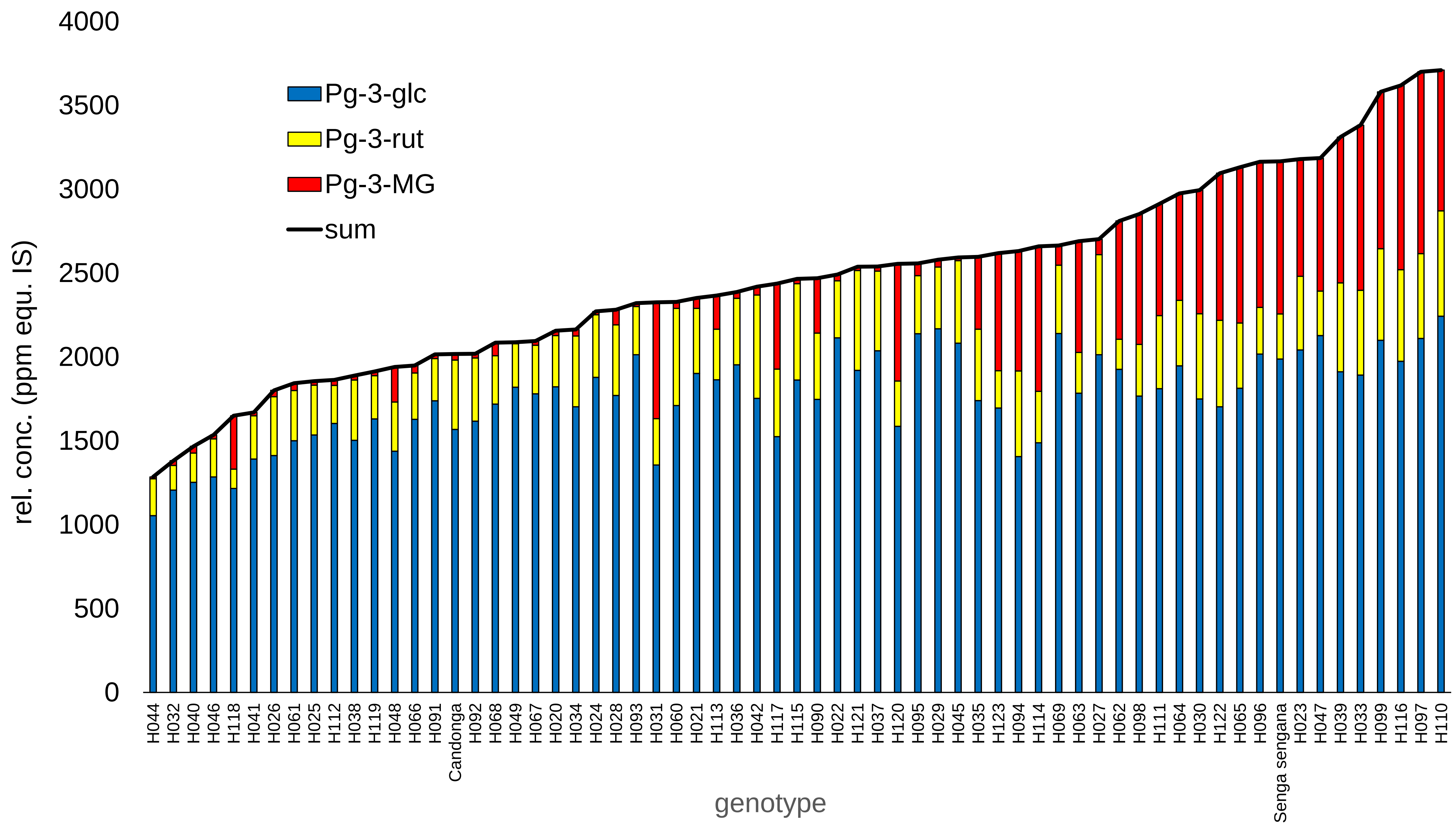

Figure S2. Formation of malonylated pelargonidin-3-glucoside facilitates the accumulation of larger amounts of pelargonidin metabolites. Pelargonidin-3-glucoside (Pg-3-glc), pelargonidin-3-rutinoside (Pg-3-rut) and pelargonidin-3-(6'-malonyl)glucoside (Pg-3-MG) were quantified relative to an internal standard (IS) by LC-MS in fruits of the GoodBerry population. The progeny fruits that produced the highest amounts of pelargonidin metabolites contained the highest concentration of Pg-3-MG. H044, H061, H092, H025, H041, and H038 are inbred lines of 'Candonga‘.

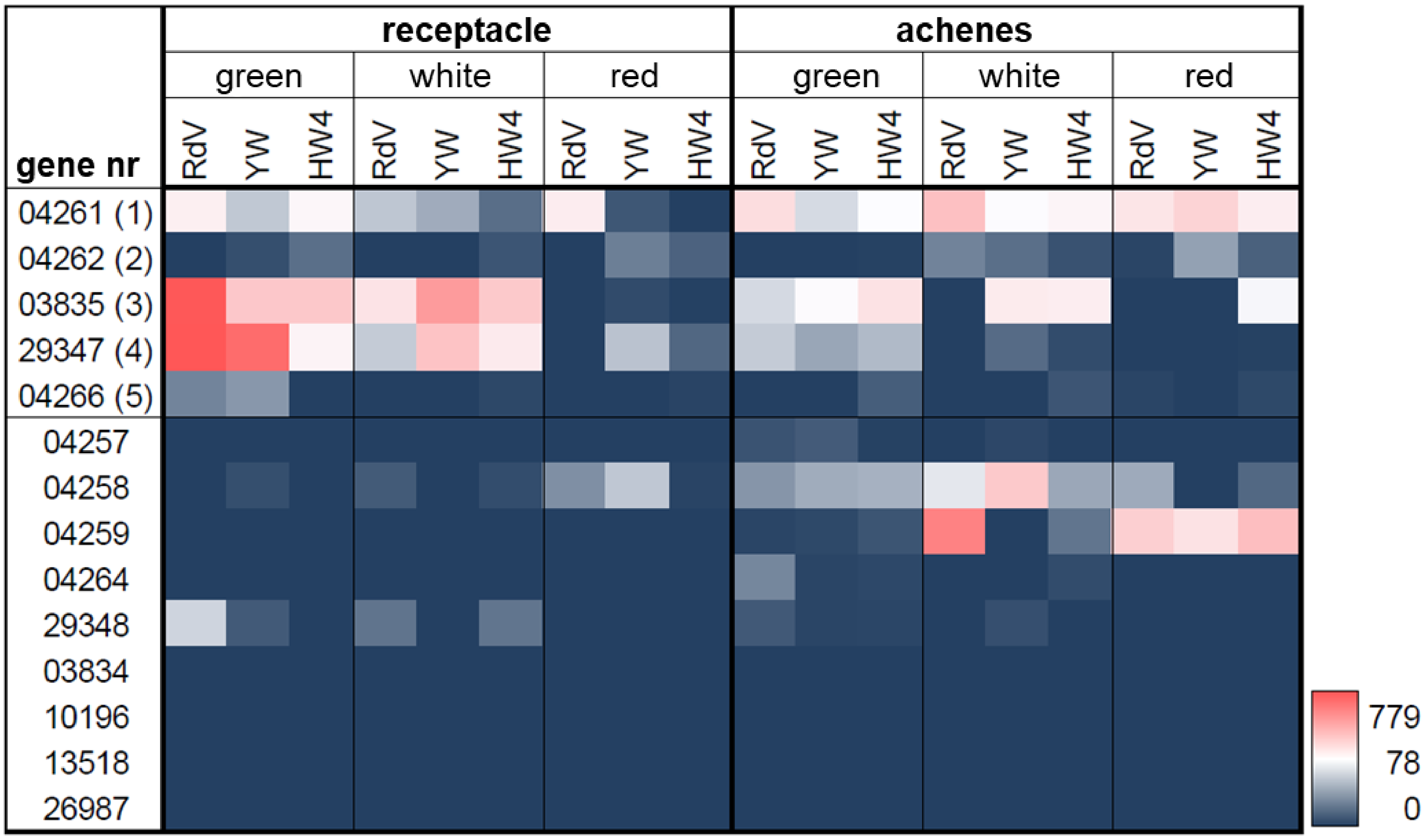

Figure S3. Heatmap of the transcript levels of *MAT* candidate genes in *Fragaria vesca* cultivars. Rdv: 'Reine des Vallées', YW: 'Yellow Wonder', HW4: 'Hawaii 4' (Härtl *et al*., 2017). Transcript sets are shown as number of transcripts per million mapped transcripts. Minimum 0 in blue and maximum 779 in red.

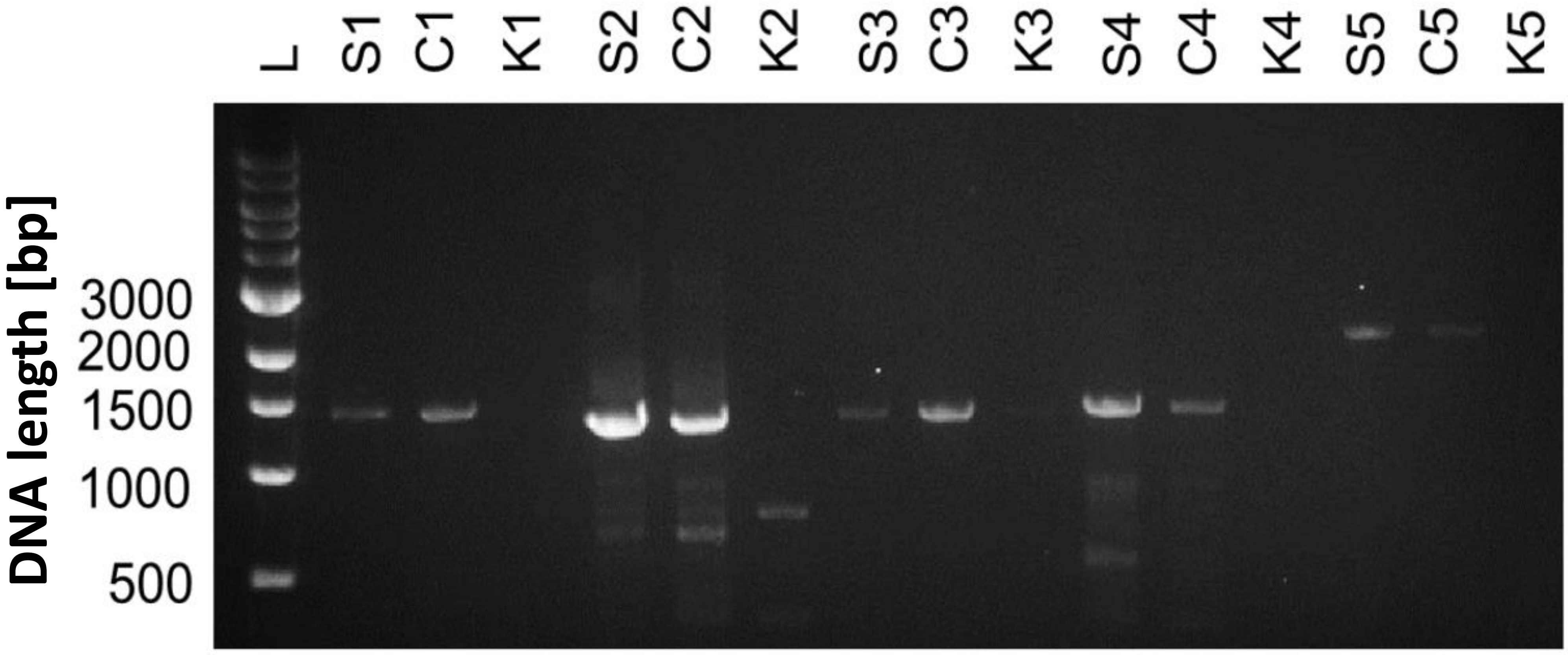

Figure S4. Agarose gel of the amplified MAT candidate genes. L - DNA molecular weight ladder, S1-S5 - DNA amplicons from 'Senga Sengana', C1-C5 - DNA amplicons from 'Candonga' , K1-K4 - negative controls

A

Gene ID	FaMAT	DNA sequence idenity	Protein sequence idenity	Difference in protein sequence
04261	1	99.4%	99.4%	A109T; 243: -V; A291G
04262	2	96.9%	80.8%*	Difference especially between 279-346; stop codon in 'Senga Sengana' sequence at 335
03835	3	100%	100%	identical
29347	4	98.4%	97.9%*	10 aa exchanges including a stop codon at position 47 in the 'Senga Sengana' sequence
04266	5	99.7%	99.6%	V330L; V348I

B

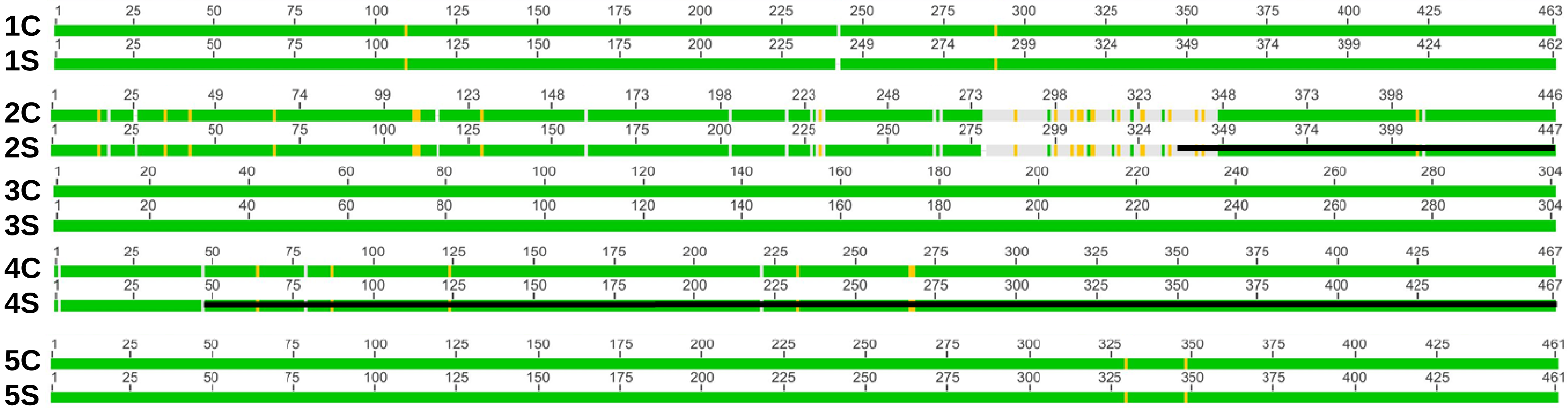

Figure S5. *FaMAT* candidates from *F. x ananassa* 'Candonga' and ‘Senga Sengana‘ selected based on a transcriptome analysis of *F. vesca* varieties. (**A**) DNA and protein sequence identity of *FaMAT* candidates. (**B**) Protein sequence alignment of the candidates FaMAT1 to FaMAT5. C1 to C5 and S1 to S5 represent the respective allelic forms from 'Candonga' and 'Senga Sengana': green - identical, yellow - similar, gray - not similar, black – non-coded region. Two of the ten sequences showed a premature stop codon, represented by a black bar.

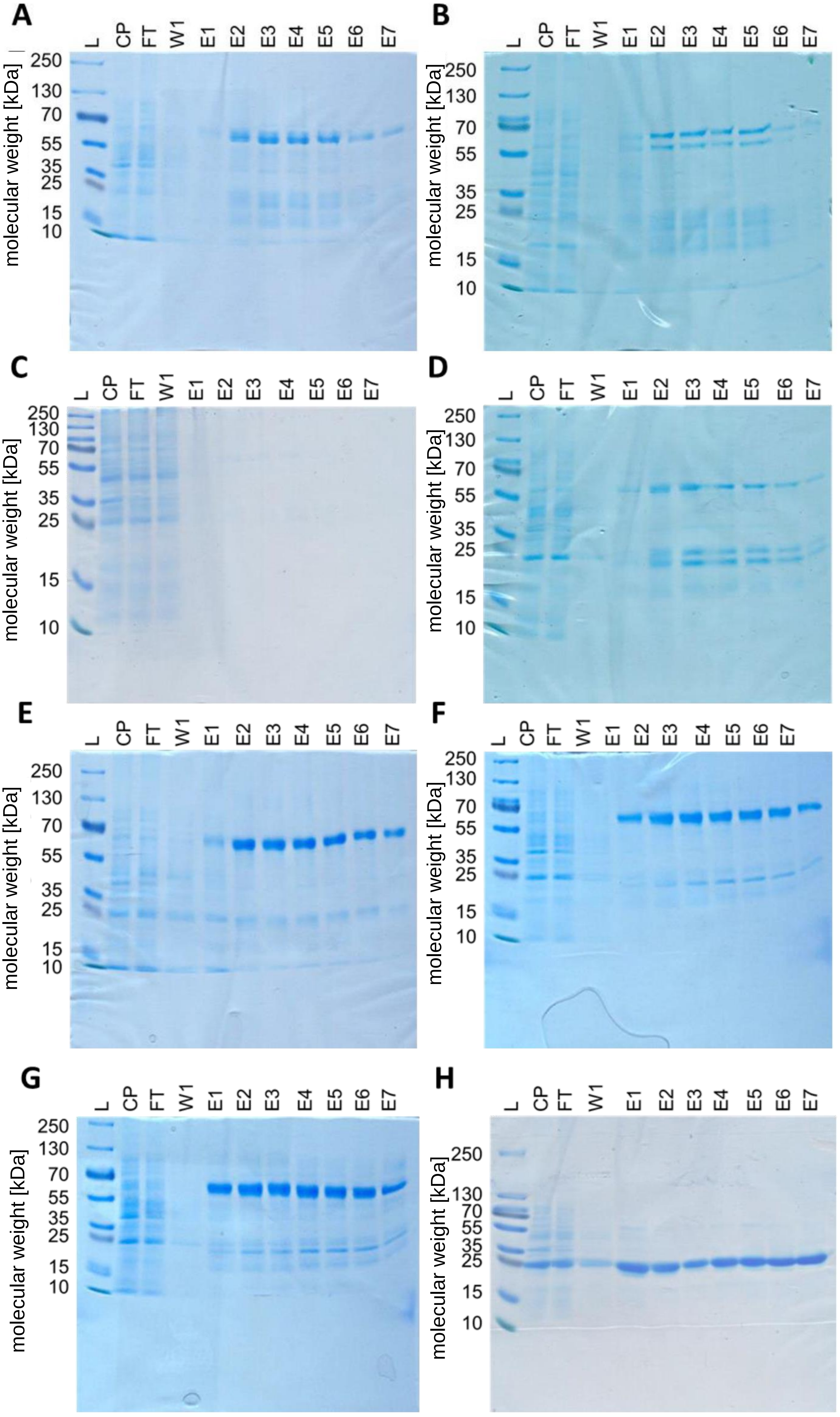

Figure S6. SDS-PAGE analysis of recombinant MAT proteins. L - protein molecular weight ladder, CP -crude protein, FT - flow through, W1 - wash fractions 1, E1-7 - protein eluates 1-7; A: S1, B: C1, C: C2, D: C3, E: C4, F: C5, G: S5, H: Empty Vector control

A

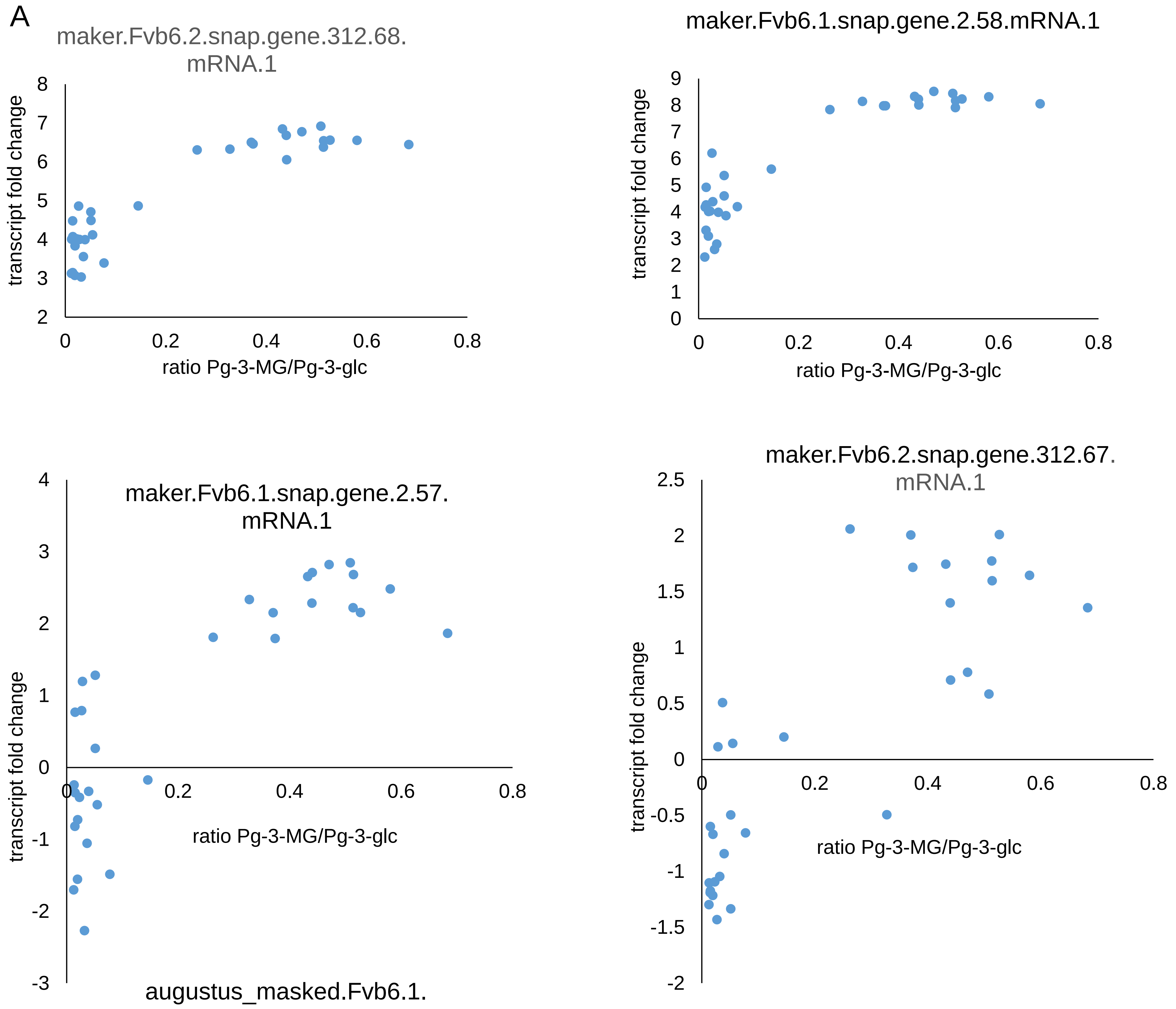

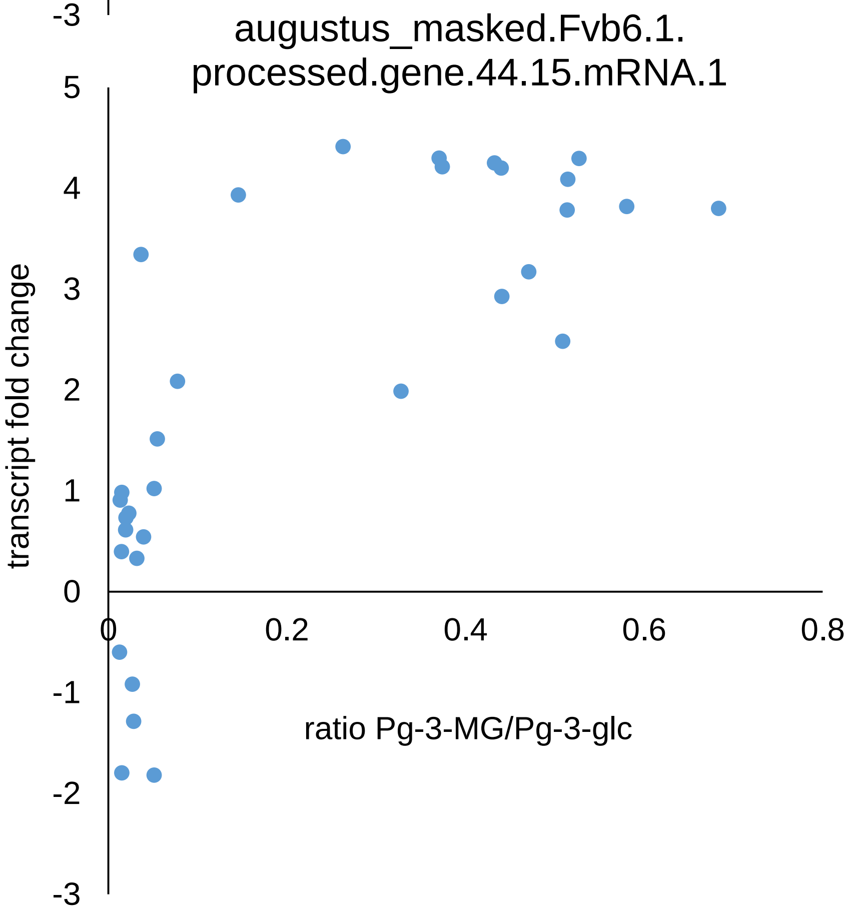

B

Transcript candidate Nr.	Transcript ID	Correlation coefficient	number of nucleotides
1	maker.Fvb6.2.snap.gene.312.68.mRNA.1	0.91	13,824
2	maker.Fvb6.1.snap.gene.2.58.mRNA.1	0.90	5,205
3	maker.Fvb6.1.snap.gene.2.57.mRNA.1	0.84	1,338
4	maker.Fvb6.2.snap.gene.312.67.mRNA.1	0.83	1,539
5	augustus_masked.Fvb6.1.processed.gene.44.15.mRNA.1	0.77	1,383

C

Transcript ID	'Candonga'	'Senga Sengana'
maker-Fvb6-2-snap-gene-312.68-mRNA-1	2.5	14.3
maker-Fvb6-1-snap-gene-2.58-mRNA-1	4.0	45.9
maker-Fvb6-1-snap-gene-2.57-mRNA-1	0.5	4.0
maker-Fvb6-2-snap-gene-312.67-mRNA-1	0.2	0.8
augustus_masked-Fvb6-1-processed-gene-44.15-mRNA-1	1.4	3.8

Figure S7. Correlation analysis of transcripts and the ratio of Pg-3-MG and Pg-3-glc. (**A**) Scatterplots showing the fold changes of the indicated transcripts in red versus green fruits of different GoodBerry genotypes with respect to the ratio of Pg-3-MG/Pg-3-glc. (**B**) Table summarizing the respective correlation coefficients. (**C**) Transcripts per million (TPM) of the indicated transcripts for 'Candonga' and 'Senga Sengana'.

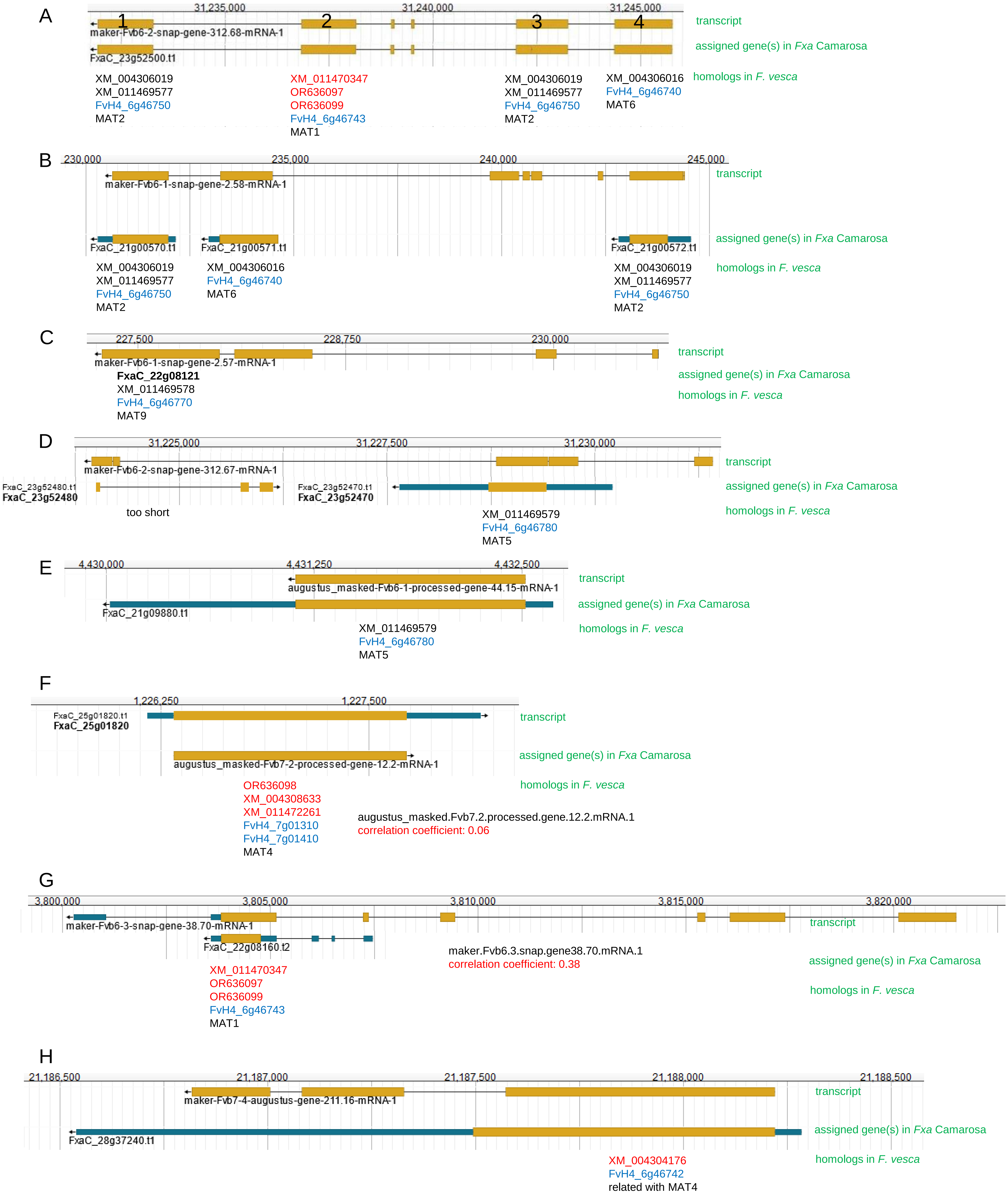

Figure S8. Excerpts from the Genome Database for Rosaceae (www.rosaceae.org). Sequences of the candidate transcripts and assigned genes. (**A**) maker.Fvb6.2.snap.gene.312.68.mRNA.1; (**B**) maker.Fvb6.1.snap.gene.2.58.mRNA.1; (**C**) maker.Fvb6.1.snap.gene.2.57.mRNA.1; (**D**) maker.Fvb6.2.snap.gene.312.67.mRNA.1; (**E**) augustus_masked.Fvb6.1.processed.gene.44.15.mRNA.1; (**F**) augustus_masked.Fvb7.2.processed.gene.12.2.mRNA.1; (**G**) maker.Fvb6.3.snap.gene38.70.mRNA.1. (**H**) maker.Fvb7.4.augustus.gene.211.16.mRNA.1. Orange coloured bars indicate suspected exons. BLAST searches showed that some transcripts contain more than one MAT sequence. The most similar sequences found in the BLAST search are listed under the respective sequence segments. Nine MAT groups were defined based on sequence similarity (highlighted in blue colour).

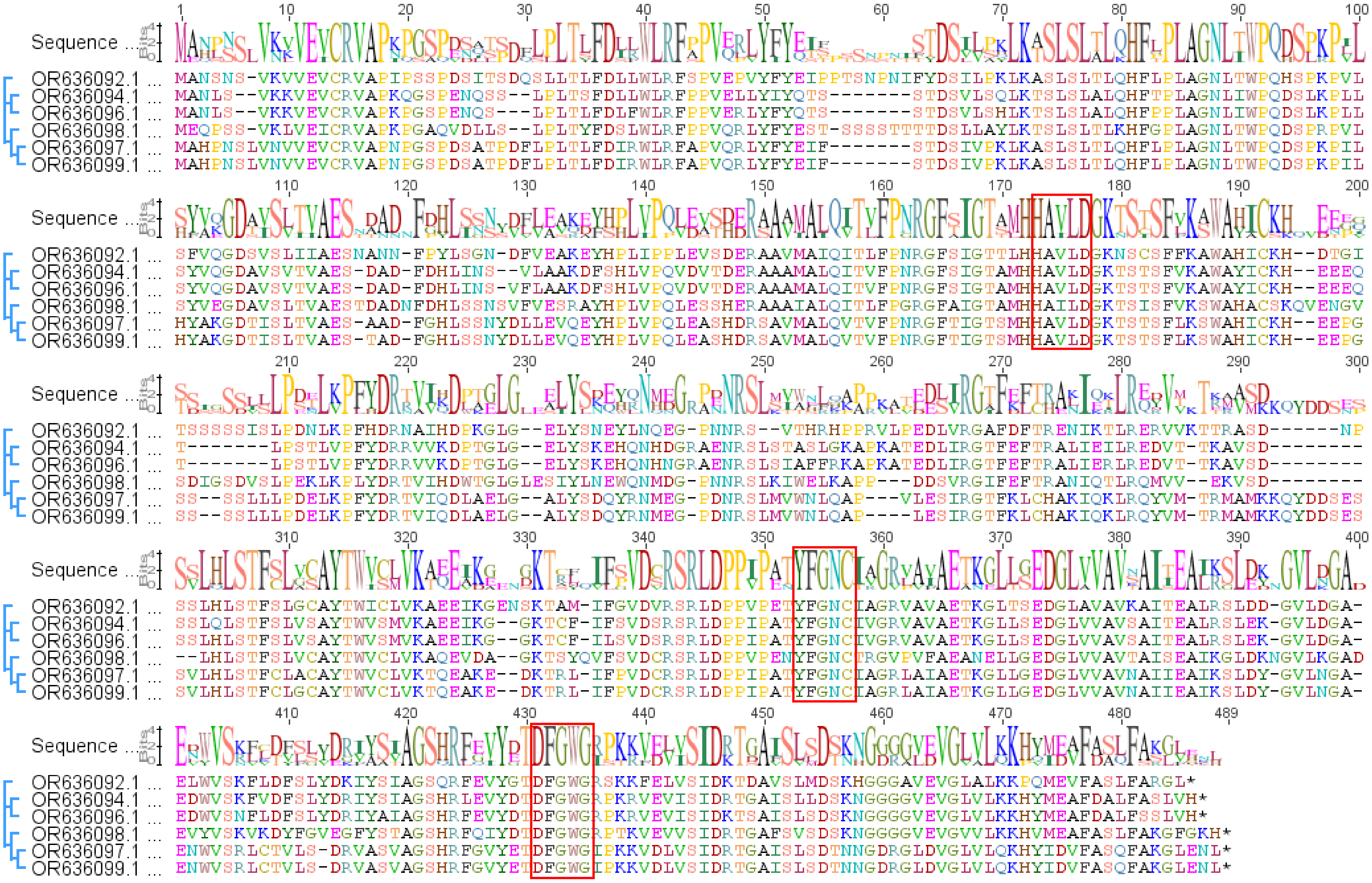

Figure S9. Protein sequence analysis of enzymatically active MAT enzymes. The alignment was performed by Geneious Basic 5.6.7 with default values. For sequence identification refer to Supplemental information Table S3). The sequences show the typical motifs HXXXD and DFGWG of the BAHD enzyme family and the YFGNC motif of anthocyanin acyl-Co transferases

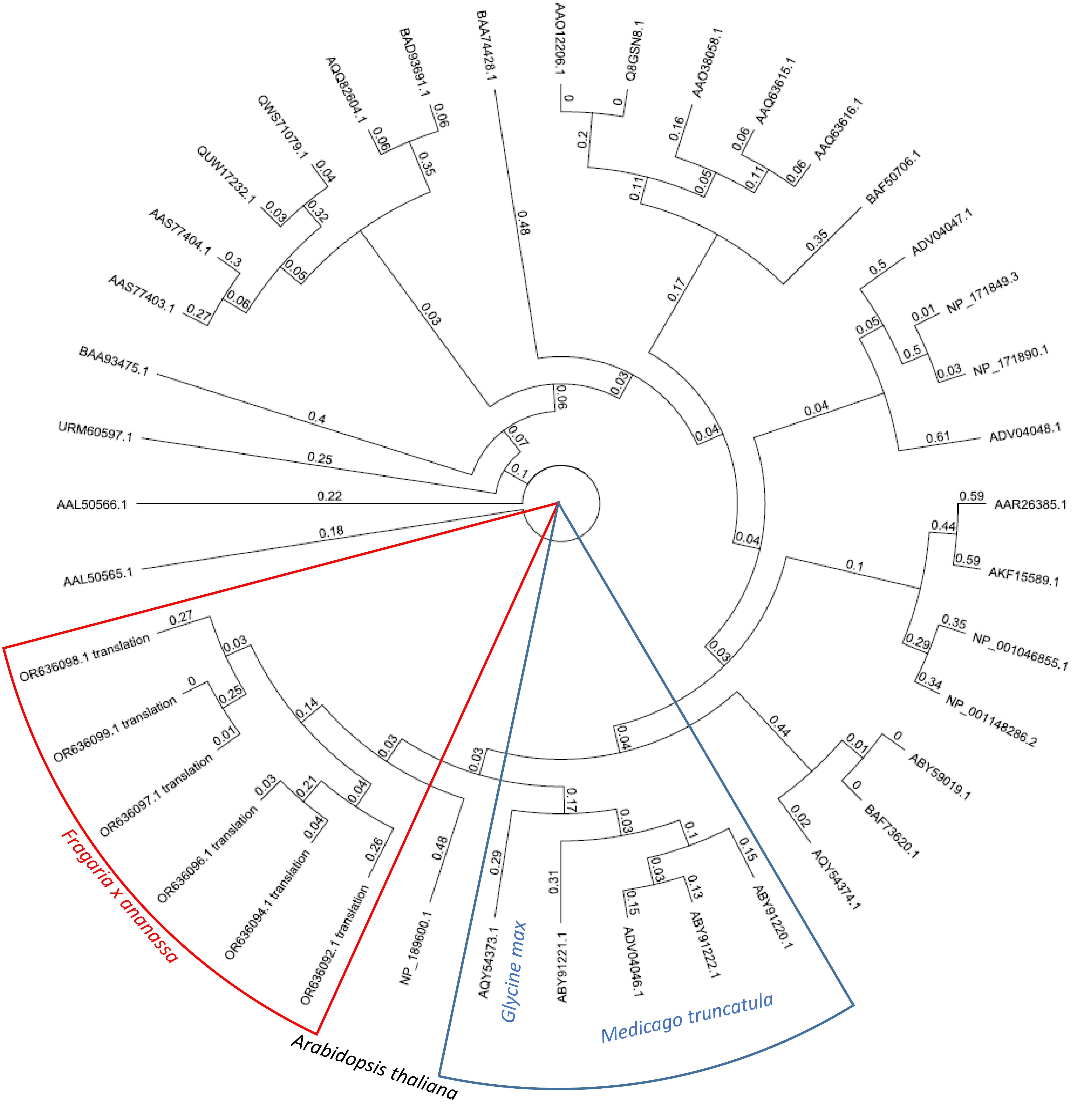

Fig. S10. Phylogenetic analysis of the protein sequences of functional MAT enzymes from different plant species. Sequences were aligned with Geneious 5.6.7 using the Geneious alignment tool with default values. The tree was built by the Neighbor-Joining method with no outgroup and 1000 replicates. The genetic distance model was Jules-Cantor. The numbers indicate substitutions per site at amino acid level. The following proteins were used to create the tree.

URM60597.1 malonyltransferase [Cistanche tubulosa], BAA93475.1 anthocyanin acyltransferase, partial [Perilla frutescens], AAL50565.1 malonyl CoA:anthocyanin 5-O-glucoside-6'''-O-malonyltransferase [Perilla frutescens], AAL50566.1 malonyl CoA:anthocyanin 5-O-glucoside-6'''-O-malonyltransferase [Salvia splendens], BAA74428.1 Anthocyanin 5-aromatic acyltransferase [Gentiana triflora], BAF50706.1 anthocyanin malonyltransferase homolog [Chrysanthemum x morifolium], Q8GSN8.1 malonyl-coenzyme A:anthocyanin 3-O-glucoside-6''-O-malonyltransferase [Dahlia pinnata], AAO12206.1 malonyl CoA:anthocyanin 3-O-glucoside-6''-O-malonyltransferase [Dahlia pinnata], AAO38058.1 malonyl-coenzyme A: anthocyanidin 3-O-glucoside-6''-O-malonyltransferase [Pericallis cruenta], AAQ63615.1 anthocyanidin 3-O-glucoside-6''-O-malonyltransferase [Chrysanthemum x morifolium], AAQ63616.1 anthocyanidin 3-O-glucoside-3'',6''-O-dimalonyltransferase [Chrysanthemum x morifolium], ADV04047.1 malonyl CoA:flavonoid malonyltransferase 5 [Medicago truncatula], NP_171890.1 HXXXD-type acyl-transferase family protein [Arabidopsis thaliana], NP_171849.3 HXXXD-type acyl-transferase family protein [Arabidopsis thaliana], NP_189600.1 malonyl-CoA:anthocyanidin 5-O-glucoside-6''-O-malonyltransferase [Arabidopsis thaliana], AAS77403.1 quercetin 3-O-glucoside-6''-O-malonyltransferase [Glandularia x hybrida], AAS77404.1 quercetin 3-O-glucoside-6''-O-malonyltransferase [Lamium purpureum], AQQ82604.1 malonyltransferase [Nicotiana benthamiana], BAD93691.1 malonyltransferase [Nicotiana tabacum], ABY59019.1 malonyltransferase MT7 [Glycine max], BAF73620.1 malonyl-CoA:isoflavone 7-O-glucoside-6''-O-malonyltransferase [Glycine max], AQY54373.1 isoflavone malonyltransferase IMaT1 [Glycine max], AQY54374.1 isoflavone malonyltransferase IMaT3 [Glycine max], ABY91220.1 isoflavonoid malonyl transferase 1 [Medicago truncatula], ABY91222.1 isoflavonoid malonyl transferase 2, partial [Medicago truncatula], ABY91221.1 isoflavonoid malonyl transferase 3 [Medicago truncatula], ADV04046.1 malonyl CoA:flavonoid malonyltransferase 4 [Medicago truncatula], ADV04048.1 malonyl CoA:flavonoid malonyltransferase 6 [Medicago truncatula], NP_001148286.2 transferase [Zea mays], NP_001046855.1 Os02g0483500 [Oryza sativa Japonica Group], AAR26385.1 anthocyanin 5-O-glucoside-4'''-O-malonyltransferase [Salvia splendens], AKF15589.1 anthocyanin acyltransferase [Vitis vinifera], QUW17232.1 BAHD-type malonyltransferase 1 [Digitalis lanata], QWS71079.1 BAHD-type malonyltransferase 2 [Digitalis lanata]

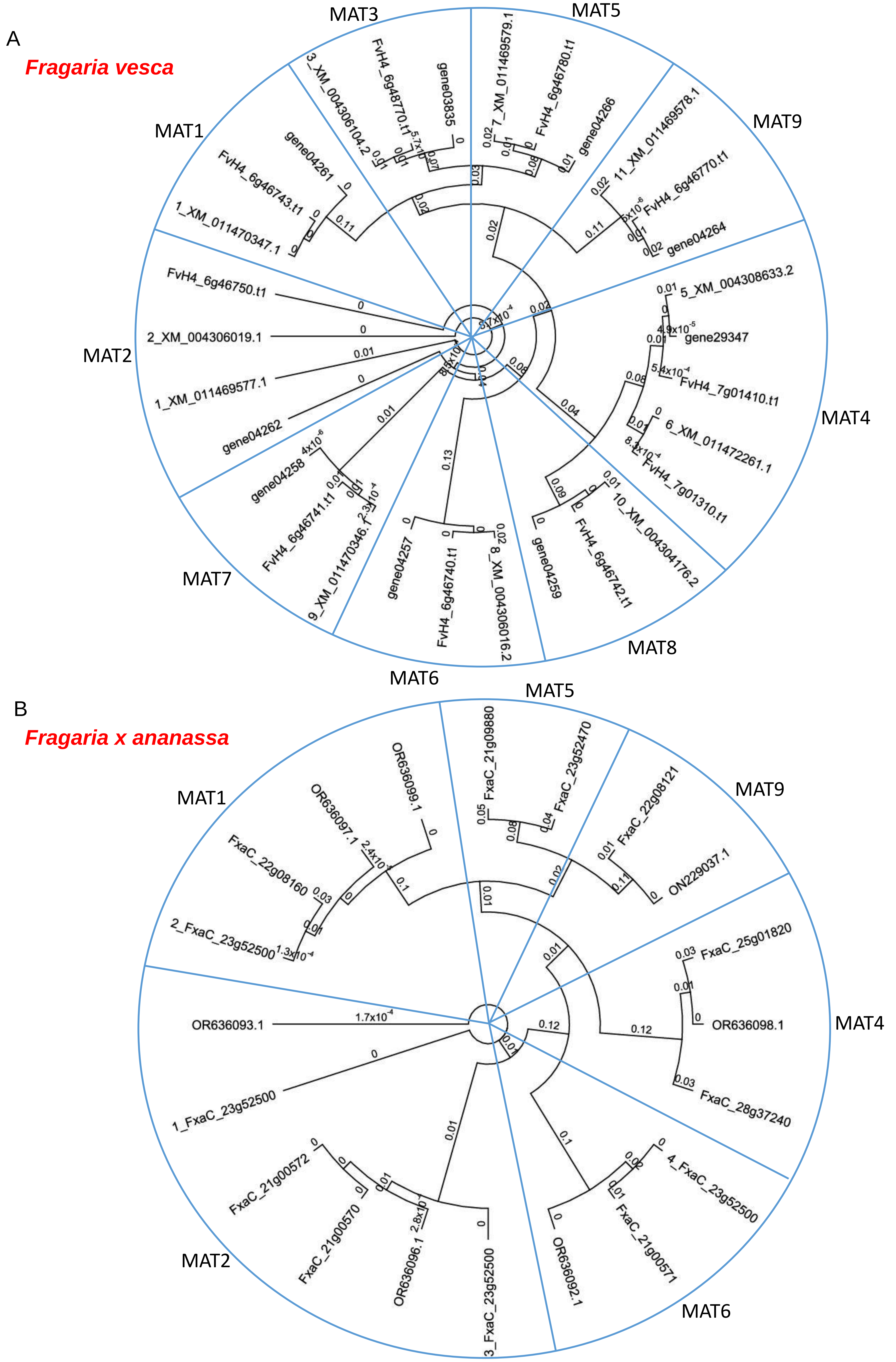

Figure S11. Phylogenetic analysis of the nucleotide sequences of selected MAT genes from different Fragaria species. (**A**) MAT1 to MAT9 from *Fragaria vesca* according to Table S3. (**B**) The corresponding MAT1, 2, 4, 5, 6, and 9 genes from *Fragaria x ananassa* according to Table S3. Sequences were aligned with Geneious 5.6.7 using the Geneious alignment tool with default values. The tree was built by the Neighbor-Joining method with no outgroup and 1000 replicates. The genetic distance model was Jules-Cantor. The numbers indicate substitutions per site at nucleotide level.

Supplementary Methods

Methods S1. LC-MS system

HPLC System
Agilent 1100 Series (Agilent Technologies, Santa Clara, CA, USA)

Pump	Quaternary pump G1311A (Agilent)
Injector	Sample injector G1313A (Agilent)
Injection Volume	5 µl
Column	Luna 3 µ C18(2) 100 Å, 150 ×2 mm (Phenomenex, Aschaffenburg)
Pre-column	Security Guard Cartridges C18 4 ×2 mm (Phenomenex)
Column Temperature	25 °C
Mobile Phase	A: 0.1% formic acid in water<br>B: 0.1% formic acid in methanol
Flow Rate	0.2 ml/min
Gradient 1 (Polyphenols)	0 – 50% B in 30 min<br>50 – 100% B in 5 min<br>100% B for 15 min<br>100 – 0% B in 5 min<br>0% B for 10 min<br>Total runtime: 65 min
Gradient 1 (FaMAT Assay)	10 – 50% B over 7 min<br>50 – 100% B in 3 min<br>100% B for 5 min<br>100 – 10% B in 5 min<br>10% B for 10 min<br>Total runtime: 30 min
DAD	Diode Array Detector G1315B (Agilent)<br>190–600 nm

coupled with

MS System
Bruker Daltonics esquire 3000plus Ion Trap (Bruker Daltonics, Bremen, Germany)

Spray Gas	Nitrogen (30.0 psi)
Drying Gas	Nitrogen (330 ° C, 9 l/min)
Resolution	13000 m/z/s
Scan Range	*m/z* 50 – 975
Polarity	alternating positive/negative
ICC Target	30000 (positive) or 10000 (negative)
Max. Accumulation Time	200 ms
Target Mass (SPS)	m/z 400
Capillary Voltage	± 4000 V
End Plate Voltage	± 3500 V
MS/MS	Auto-tandem MS
Collision Gas	Helium 5.0 (4.21 × 10^{-6} mbar)
Collision Energy	1.0 V
Skimmer	± 40.0 V
Cap Exit	± 121 V
Oct 1 DC	± 12.0 V
Oct 2 DC	± 1.7 V
Trap Drive	45.6
Oct RF	112.2 Vpp
Lens 1	± 5.0 V
Lens 2	± 60.0 V
